# Soil lead, zinc, and copper in two urban forests as influenced by highway proximity

**DOI:** 10.1002/jeq2.20642

**Published:** 2024-10-21

**Authors:** Maryam Foroughi, Raymond R. Weil

**Affiliations:** ^1^ Environmental Science & Technology University of Maryland College Park Maryland USA

## Abstract

Heavy metals emitted by vehicles have the potential to accumulate in soil near roadways, threatening the health of soil, plants, animals, and humans. This study evaluates Pb, Zn, and Cu levels in forest O‐horizons, mineral soil, and earthworms near busy roadways in the metro‐Washington, DC area. The study sites comprised road‐edge environments within urban parks. Six transects were sampled in each park, collecting mineral soil at 1‐ to 30‐m distances from the road edge and dividing it into eight depth increments (0–30 cm). O‐horizon plant litter and earthworm samples were also collected at these locations. All samples underwent total Pb, Zn, and Cu X‐ray fluorescence analysis. Generally, Pb concentrations (in upper 0–10 cm) were 1–4.8 times higher at 3 m compared to 30 m from the road, with less consistent gradients for Zn and Cu. Concentrations peaked near the soil surface, with lower levels in the O‐horizon above and deeper soil layers. Leaded vehicle fuel was phased out by the early 1980s, but legacy Pb contamination persisted in roadside forests, averaging 365 mg kg^−1^ in the upper 10 cm within 3 m of the roadway (< EPA action level of 1200 mg kg^−1^ for non‐play areas). Zinc, often present in vehicle tires, accumulated in earthworms to 192–592 mg kg^−1^, concentrations exceeding those in the soil, while Pb and Cu were less concentrated in earthworms than in either O‐horizon or mineral soil. Factors such as plant uptake, erosion, wind, soil texture, and metal solubility influence how heavy metals redistribute and bioaccumulate in the O‐horizon, mineral soil, and soil fauna.

## Core Ideas

- Roadside heavy metals pose threats to soil, plant, and animal health.
- Despite fuel changes, legacy lead remains a concern.
- Ecological repercussions are indicated by zinc accumulation in earthworms.
- Environmental management benefits from understanding metal redistribution.
- Ongoing monitoring for ecosystem preservation is necessary due to vehicular emissions.

**Abbreviations**
BSAF: biota‐to‐soil accumulation factor
CEC: cation exchange capacity
NACE: National Capital Parks‐East
ROCR: Rock Creek National Park
SOM: soil organic matter
XRF: X‐ray fluorescence

## 1 INTRODUCTION

Heavy metals (i.e., lead, copper, and zinc) from fuel, brake linings, tire wear, and oil leaks may spread in aerial suspension to roadside land (Awofolu, 2004; Hansmann & Köppel, 2000) where they can contaminate soil, plants, and animals (Abdelhafez et al., 2015; Lone et al., 2008; Möller et al., 2005). Vehicular traffic is a major source of heavy metals in the air near roadways (Chrzan, 2013; Zereini et al., 2005). Airborne metal contaminants (Awofolu, 2004) can affect soils, plants, animals, and humans (Abdelhafez et al., 2015; Lone et al., 2008; Möller et al., 2005) and may be passed up the food web and accumulate in certain animals (Ward et al., 1974).

Tetraethyl lead was used to improve engine performance in the United States from the 1920s to the 1990s. Gasoline during the leaded gasoline phase out (1960s–1970s) contained ∼600 mg Pb/L. (Kovarik, 2005). Lead was the first and most thoroughly investigated heavy metal pollutant in roadside soil with the accumulation of Pb in roadside soil reported to relate to distance from the road and average daily traffic volume in the 1970s (Lagerwerff & Specht, 1970; Ward et al., 1975; Wheeler & Rolfe, 1970). Investigations later expanded to include Zn and Cu as airborne heavy metals (Turer et al., 2001).

Studies on forest soils in the northeastern United States (e.g., Schlesinger et al., 1974; Siccama & Smith, 1978; Turner et al., 1985) focused on metals in O‐horizons and upper mineral horizons. Human activities, particularly leaded gasoline use, caused a fivefold to 10‐fold increase in Pb concentrations in forest O‐horizons, impacting soil microbial activity (Ekenler & Tabatabai, 2002; Friedland et al., 1986; Tyler, 1976). Heavy metals deposition has been linked to decreased abundance of soil decomposer animals, decreased diversity of animal groups, and changes in soil microflora (Bååth, 1989; Tyler et al., 1989). High heavy metal concentrations dramatically altered understory vegetation (Salemaa & Uotila, 2001) and reduced forest stands and tree growth rates near pollution sources (Mälkönen et al., 1999).

Airborne heavy metals settle on the tree foliage and the forest floor and accumulate in both the O‐horizons and upper mineral soil horizons, influenced by factors like particle size, rainfall, surface runoff, and wind patterns (Birch & Scollen, 2003; Trombulak & Frissell, 2000; Wong et al., 2006). For example, Piron‐Frenet et al. (1994) demonstrated lead pollution dispersing up to 500 m from the road source through wind during dry conditions, while in wet conditions, lead infiltrated deeper into the soil layers. Gish and Christensen (1973) reported that Pb concentration in the upper 2.5 cm of soil decreased by over 600 mg kg^−1^ from soil adjacent to the Washington–Baltimore Parkway to 48 m distant. Additionally, lead concentrations in earthworms decreased from 331 to 67 mg kg^−1^ with distance from the road. (Gish & Christensen, 1973).

Earthworms can inhabit contaminated areas and accumulate heavy metals (Cd, Cu, Pb, and Zn) from contaminated soil (Beyer et al., 1982; Martin, 2012; Morgan & Morgan, 1988), playing a vital role in bioavailability of heavy metals in soil (Ardestani et al., 2019; Wang et al., 2017). The burrowing activity of earthworms enhances soil permeability, influencing the rate and pathways of leaching, thereby impacting the mobilization rates of sorbed metals. Certain earthworm species may ingest heavy metals near the soil surface and transport them to deeper soil layers (Dragicevic et al., 2017). Previous research reported the concentrations of Cd, Pb, and Zn in earthworms galleries were greater than those in the corresponding bulk soils at the same depth (Sterckeman et al., 2000). Thus, particles and solutes may move with water through the earthworm's galleries and transport Pb and other metals to deeper soil layers (Sterckeman et al., 2000; Trojan & Linden, 1994).

Unlike Pb, Cu and Zn have garnered less long‐term study. As essential nutrients for plants and animals, they cycle through soil and plants. Elevated Cu and Zn levels can be phytotoxic, whereas Pb is typically refused by plant roots except in rare cases (Johnson & Richter, 2010). Limited research exists on the individual and combined effect of discontinuing Pb in gasoline and Cu restrictions in brake linings on the spatial and temporal distribution of these heavy metals in forest soils, plants, and soil fauna along high‐traffic roads.

This study aimed to evaluate the combined impact of historical and ongoing heavy metals emissions from vehicles and road surfaces on forest soils, including earthworm populations, along two heavily trafficked roads in the Washington, D.C. metro area. We hypothesized that because of ongoing Zn and Cu emissions, the concentrations of these metals would decrease with soil depth. However, we anticipated that Pb concentrations, due to the cessation of emissions nearly 35 years ago, might peak a few centimeters below the current soil surface (A‐horizon) due to limited plant cycling and burial by freshly deposited mineral soil from upslope erosion. Additionally, we hypothesized concentrations of all three metals in mineral soil, leaf litter, and earthworms to decrease logarithmically with distance from the road.

**Core Ideas**

- Roadside heavy metals pose threats to soil, plant, and animal health.
- Despite fuel changes, legacy lead remains a concern.
- Ecological repercussions are indicated by zinc accumulation in earthworms.
- Environmental management benefits from understanding metal redistribution.
- Ongoing monitoring for ecosystem preservation is necessary due to vehicular emissions.

## 2 MATERIALS AND METHODS

### 2.1 Study sites

**FIGURE 1:**
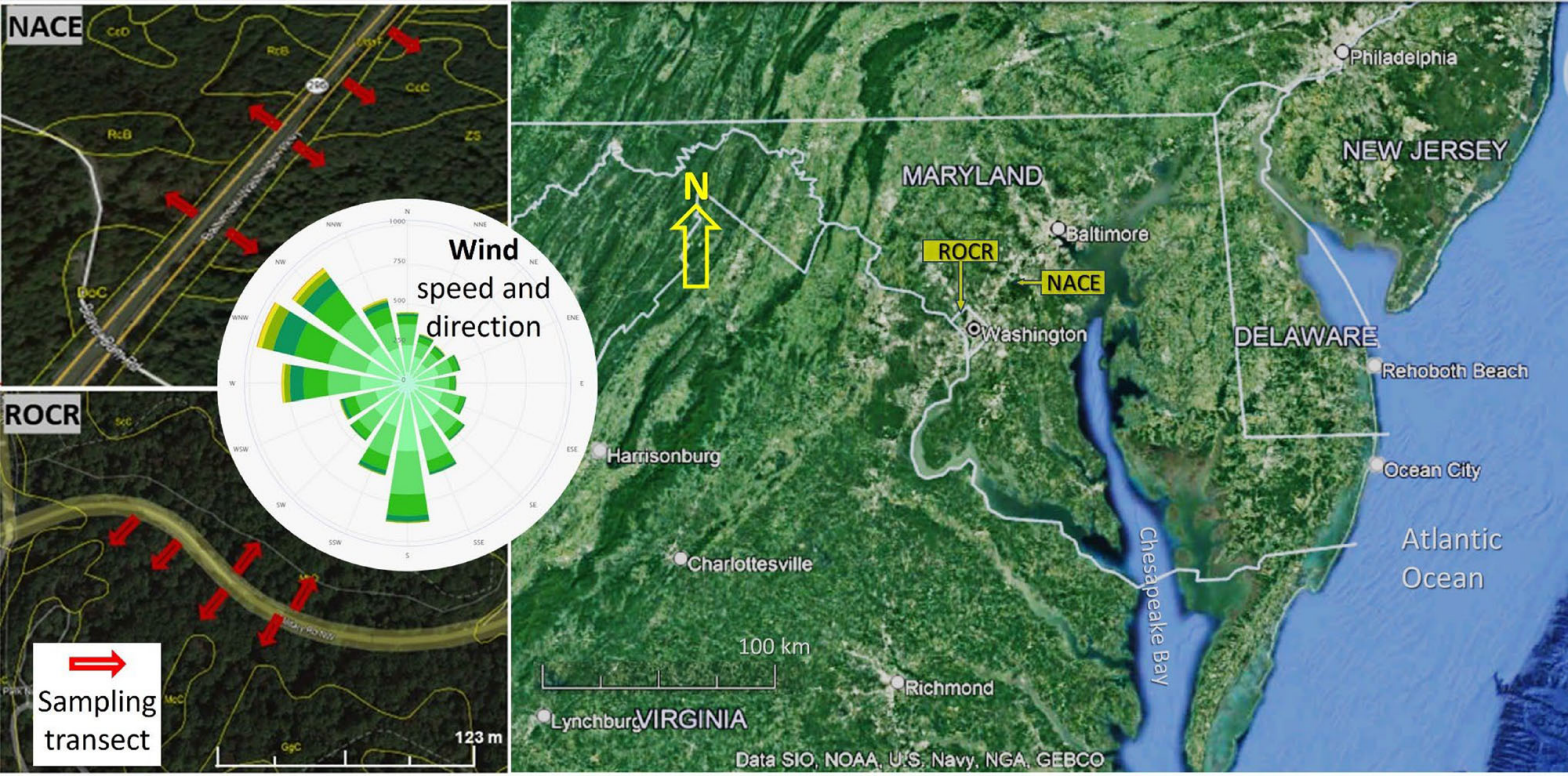
Location map for Rock Creek Park (ROCR) and National Capital Parks‐East (NACE) and the sampling transects and heavily trafficked roads therein.

The study was conducted in forested areas of Rock Creek National Park (ROCR) along Military Road in Washington, D.C., and the National Capital Parks‐East (NACE) along the Baltimore–Washington Parkway in Maryland (Figure 1). Both roads have been heavily trafficked since before 1960. Military Road was heavily trafficked since the introduction of leaded gasoline in the 1920s (Shoemaker, 1909), while the Baltimore–Washington Parkway was built circa 1950. In 1966, nearby sites along the B–W Parkway recorded soil Pb level (extracted with HCl) ranging from 300 to 540 mg kg^−1^ at a distance of 8 m from the road (Lagerwerff & Specht, 1970). Soils in ROCR are mainly moderately acidic coarse‐loamy, micaceous, mesic Typic Dystrudepts formed from gneiss rocks while those in NACE are mainly extremely acidic, fine, kaolinitic, mesic Aquic Hapludults formed from highly weathered coastal plain sediments (USDA NRCS, 2019). The forest at ROCR comprised 120‐year‐old mixed hardwood regrowth dominated by American beech (*Fagus grandifolia*), oak species (*Quercus* spp.), and tulip (*Liriodendron tulipifera*) trees (Einberger, 2014). The forest at NACE comprised 90–100‐year‐old mixed hardwood and conifer regrowth dominated by Virginia pine (*Pinus virginiana*), oak species, and black gum (Nyssa sylvatica) species (Walsh et al., 2016).

### 2.2 Sampling methods

Mineral soil and O‐horizon (organic litter) samples were collected from ROCR March 27 to April 23, 2021, and from NACE June 16 to 29, 2021, using six transects perpendicular to the roadway in each park (Figure 1). At 1, 3, 5, 7, 10, 13, 15, 20, 25, and 30 m from the paved road mineral soil cores (3.04 cm inside diameter) were collected and cut into 0–2.5, 2.5‐5, 5–7.5, 7.5‐10, 10–15, 15–20, 20–25, and 25–30 cm depth increments. To sample the O‐horizon, all plant litter in various stages of decay (except woody material > 2‐cm diameter) above the mineral soil was collected within a 625 cm^2^ square at each soil coring location. There were six replicates for the soil analysis for each soil depth at each distance in ROCR and NACE. Samples from these replicates were analyzed in triplicate to ensure accuracy. Between October 16 and November 09, 2021, earthworms were sampled at 3, 5, 10, and 30 m from the road along six of the sampling transects in ROCR and three transects in NACE (no earthworms were observed at the other three transects in NACE, which had thick O‐horizon layers). To sample earthworms, the lose litter (Oi horizon) was removed and a 40‐cm diameter metal cylinder was inserted into the soil to a depth of 5 cm. Then 5 L of earthworm irritant solution (8 g hot mustard powder per L water) was slowly sprinkled into the cylinder and allowed to soak into the soil (Fox et al., 2017). Earthworms surfacing within 10 min were collected, rinsed with DI water, and placed in individual cups for each location. In the lab, they were kept on damp filter paper in Petri dishes without food for 3 days to eject gut soil. Subsequently, they were counted, weighed, and frozen for analysis.

### 2.3 Sample analysis

Mineral soil samples were dried at 65°C for 2 days. Soil bulk density was determined by dividing the dry mass of the soil by the volume of the soil core. The difference in soil dry weight after 65°C and 105°C drying was negligible (<0.5%). The dry mineral soil was then crushed and sieved through a 2 mm sieve. O‐horizon samples were dried at 65°C and then ground to < 2 mm.

Frozen earthworms were, weighed again, dried in an oven at 65°C for 24 h, and weighed again before crushing (<1 mm) and analyzing (Morgan & Morgan, 1988). Soil pH was determined as pH_H2O_ (Gee & Or, 2002; Peech, 1965). To measure the bioavailable P, K, Mg, and Ca, soils were extracted with the Mehlich‐3 solution (Mehlich, 1978). The cation exchange capacity (CEC) and base cation saturation of soil samples were estimated from the Mehlich‐3 extractions (Sumner & Miller, 1996). Soil organic matter (SOM) was determined by loss‐on‐ignition (Magdoff et al., 1996; Schulte & Hopkins, 1996).

**FIGURE 2:**
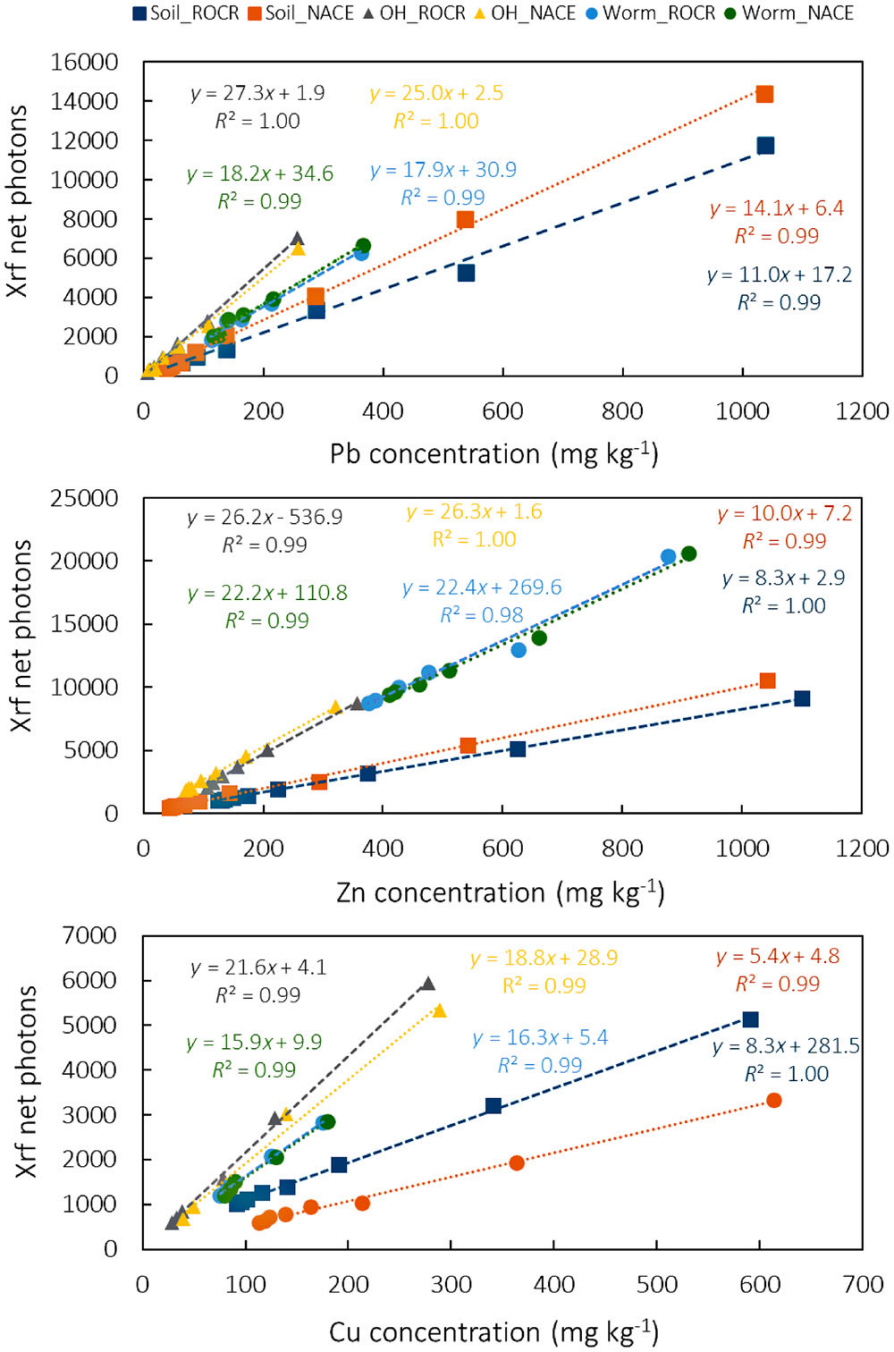
Relationships between metal concentrations and net photos detected by X‐ray fluorescence (XRF) used to calibrate the XRF data for Pb, Zn, and Cu in mineral soil (Soil), O‐horizons (OH), and earthworms (Worm).

Portable X‐ray fluorescence (XRF, Model Tracer SD3, Bruker, Billerica, MA) was used to measure heavy metals. Each sample was irradiated for 120 seconds with 40 kV, at 10.4 µA, under 10.00 keV using a filter consisting of 12 µm Al+ 1 µm Ti+ 1 µm Cu. To calibrate net photons detected to metal concentrations, we used materials (mineral soil, O‐horizon, ground earthworm tissue) spiked with a series of known additions from 5 to 1000 mg kg^−1^ of Pb, Zn, and Cu (Figure 2). The limits of detection for Pb, Zn, and Cu using the Bruker portable XRF are about 2, 1.2 and 1.4 mg kg^−1^, respectively. The precision is about ± 10%.

Linear regressions of net photons detected by XRF against Pb, Zn, and Cu concentrations in spiked samples had *R* ^2^ values >0.97, confirming XRF's ability to provide quantitative element concentrations. To make these calibration standards, we used mineral soil, O‐horizon, and earthworm samples from each park, collected 25–30 m from the road, and, for mineral soil, 25 to 30‐cm deep. Notably, calibration curves for earthworm and O‐horizon samples were consistent across both sites, particularly for Zn, whereas those for mineral soil showed significant differentiation between sites and from the organic materials (Figure 2), especially for Cu, due to variations in concentrations of other X‐ray fluorescing elements such as Ca, Mg related to the contrasting soil parent materials (Table S1). However, spectra collected at different depths indicate that the differences in Ca and Mg concentrations between shallow and deeper soils are negligible, supporting the use of samples from 25 to 30‐cm depth for all depths in the calibration process.

The metal stock (kg ha^−1^) in the mineral soil was calculated using the formula:

Stock=c.ρ.d/10

where *c* is the concentration (mg kg^−1^), *ρ* the bulk density (g cm^−3^), and d the soil depth (cm). The metal stock (kg ha^−1^) in the O‐horizon was calculated using the formula:

Stock=c.W/A/100

where *c* is the concentration of metals in the sampled organic matter (mg kg^−1^), *W* is the dry weight of the sampled organic matter (kg), and A the sample area (m^2^).

### 2.4 Statistical analysis

The Shapiro–Wilk test (dlookr R package, Ryu, 2019) was used to assess normality, and natural logarithm transformations were applied where required to satisfy assumptions of normality and homogeneity of variances and back‐transformed to present the results.

A factorial repeated measures analysis of variance was implemented in R software v4.0.2 (R Core Team, 2020) to evaluate the main effects of three factors: (direction relative to the road), distance (1–30 m from roadway), and depth (0–30 cm into soil) as well as the interactions among those factors. Repeated measurements were taken at multiple depths and distances within each transect to assess these effects. Tukey's HSD test was used for pairwise comparisons within main or interaction effects, with significance set at *p* < 0.05. The trend of Pb, Zn, and Cu concentrations with distance was investigated by log‐linear regression in the R software v4.0.2 (R Core Team, 2020). The biota‐to‐soil accumulation factor (BSAF) for Pb, Zn, or Cu relative to O‐horizon or A‐horizon (0–10 cm mineral soil) was calculated as the ratio of (concentration in earthworm)/concentration in O‐horizon or A‐horizon. Where needed to assure normality, log_10_ transformations were used. Data visualizations used SYSTAT (SYSTAT, 2022), ggplot2 (Wickham, 2016), and ggpubr (Kassambara & Kassambara, 2020) R packages.

## 3 RESULTS AND DISCUSSION

### 3.1 Mineral soil and O‐horizon Pb, Zn, Cu concentrations

**FIGURE 3:**
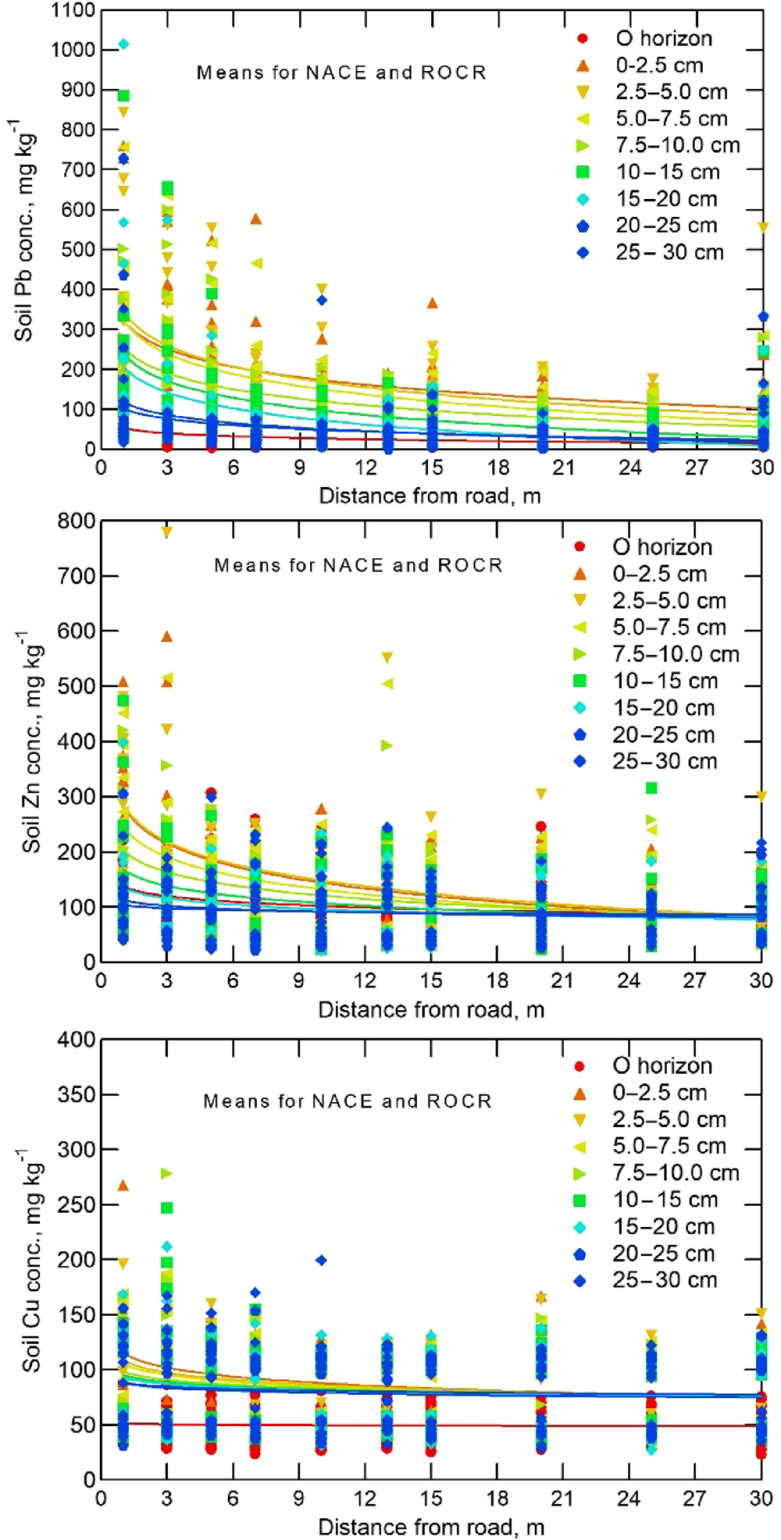
Measured and fitted (log regression lines) Pb, Zn, and Cu concentrations in mineral soil and O‐horizon material at various distances from the edge of the highway in Rock Creek Park (ROCR) and National Capital Parks‐East (NACE) for various soil depths.

**FIGURE 4:**
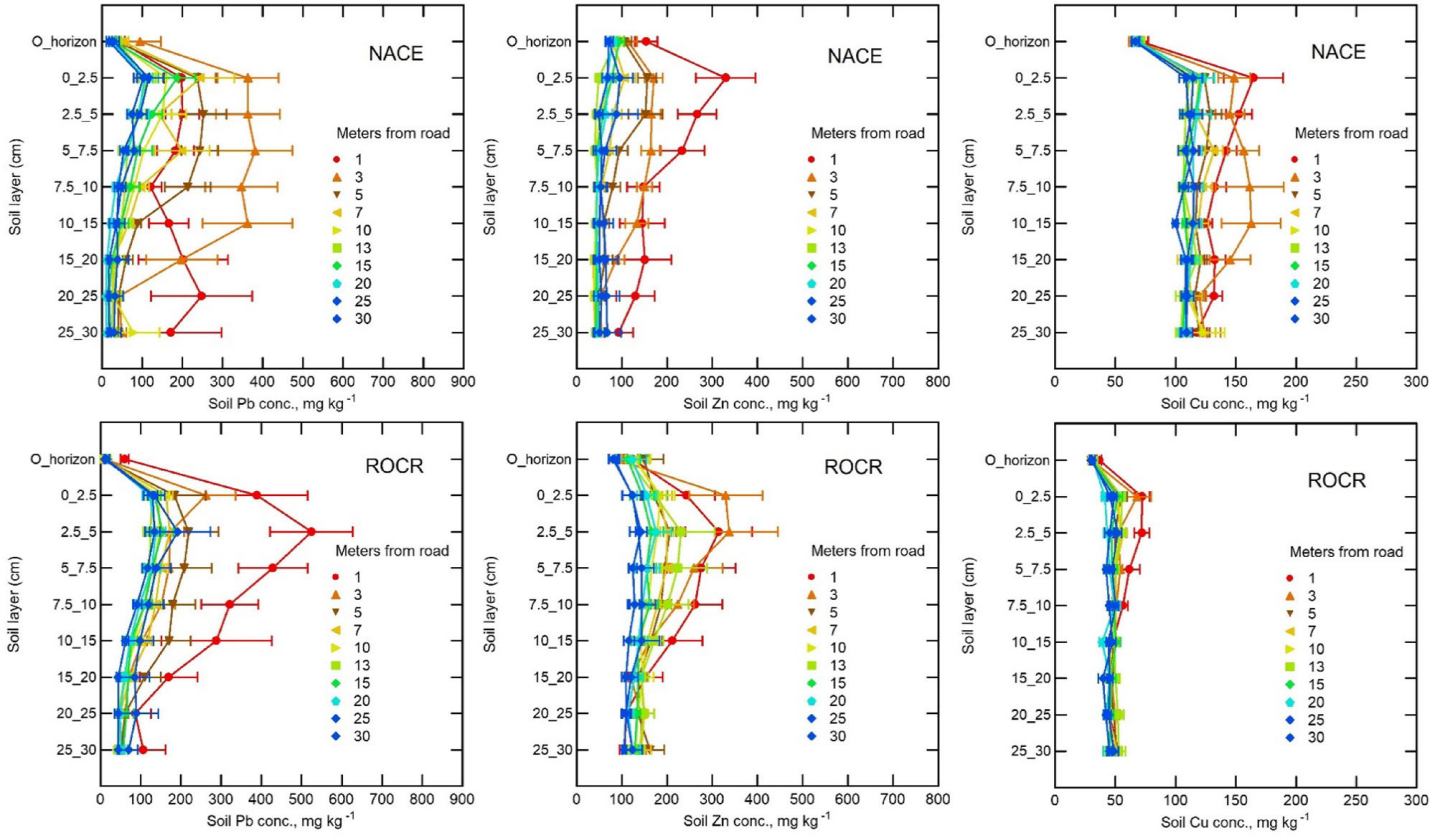
Vertical distribution of the three heavy metals throughout the soil depth at different distances from the road in Rock Creek Park (ROCR) and National Capital Parks‐East (NACE).

In all locations, the Pb concentrations generally exceeded the global normal background level (17 mg kg^−1^) for uncontaminated soils (Figure 3) (Alloway, 2012; Nriagu, 1978) and the average (61 mg kg^−1^) reported for central Maryland soils (Table 1) (Yesilonis et al., 2008). Our sampling aimed to minimize soil or dominant tree cover type variation along each transect, so significant changes in metal concentrations along the transects and with depth are likely due to historically high deposition fluxes from traffic emissions. Across all soil samples, Pb concentrations were about the same in ROCR (mean ± standard deviation: 126 ± 118 mg kg^−1^) as in NACE (109 ± 135 mg kg^−1^) (Figure 4).

**TABLE 1:** Average ± standard deviation of Pb, Zn, and Cu concentrations in A‐horizon mineral soil, O‐horizon forest floor, and earthworm dry matter at 3, 5, 10, and 30 m from the road in two National Park forests in comparison with background level.

	National Capital Parks‐East (NACE)				Rock Creek Park (ROCR)			
Distance from road (m)	O‐horizon (mg kg^−1^)	A‐horizon (0–10 cm) (mg kg^−1^)	Earthworm (*N*)	Earthworm (mg kg^−1^)	O‐horizon (mg kg^−1^)	A‐horizon (0–10 cm) (mg kg^−1^)	Earthworm (*n*)	Earthworm (mg kg^−1^)
**Pb**								
3	95.1 ± 106a	447 ± 206a	22	186 ± 57.7a	10.3 ± 3.8a	283 ± 80.4a	26	91.4 ± 73.9a
5	35.5 ± 19.8a	279 ± 105ab	17	84.6 ± 15.8ab	9.36 ± 3.0a	276 ± 204a	29	55.5 ± 42.6a
10	34.9 ± 22.1a	164 ± 69.5b	5	70.0 ± 16.2ab	8.06 ± 1.7a	188 ± 70.2a	31	43.4 ± 26.1a
30	30.8 ± 26.0a	93.6 ± 34.4b	6	23.5 ± 19.2b	14.7 ± 13.3a	200 ± 117a	17	32.4 ± 15.0a
Average	48.4 ± 43.5A	246 ± 104A	–	90 ± 27.2A	10.6 ± 5.5B	237 ± 118A	–	58.0 ± 39.4B
Background^a,b^	1–10	45–61	–	0.24–0.8	1–10	45–61	–	0.24–0.8
**Zn**								
3	114 ± 38.9a	218 ± 43.6a	22	441 ± 81.2a	86.7 ± 28.6a	335 ± 74.8a	26	525 ± 671a
5	105.1 ± 32.7a	162 ± 51ab	17	466 ± 230a	149 ± 87.4a	282 ± 48.2ab	29	457 ± 307a
10	92.8 ± 20.2a	74.8 ± 21.2b	5	592 ± 187a	128 ± 71.0a	318 ± 130ab	31	301 ± 199a
30	71.8 ± 17.5a	104 ± 78.1b	6	236 ± 118a	83.4 ± 23.2a	186 ± 35.8b	17	192 ± 97.3a
Average	95.9 ± 25.1A	140 ± 48.5B	–	434 ± 154A	112 ± 52.6A	280 ± 72.2A	–	369 ± 319A
Background^b,c^	10–150	73–100	–	152–330	10–150	73–100		152–330
**Cu**								
3	66.6 ± 10.6a	212 ± 34.8a	22	75.1 ± 25.8a	30.5 ± 1.4a	84.5 ± 14.3a	26	63.2 ± 25.1a
5	66.2 ± 6.2a	174 ± 20.2ab	17	82.5 ± 35.1a	29.7 ± 1.1a	69.1 ± 7.5ab	29	56.1 ± 9.9a
10	67.3 ± 6.2a	158 ± 9.2b	5	50.0 ± 2.2a	30.7 ± 3.1a	69.8 ± 5.1ab	31	63.3 ± 26.1a
30	66.86 ± 6.6a	154 ± 18.9b	6	48.8 ± 11.8a	30.9 ± 4.4a	67.5 ± 4.0b	17	55.2 ± 9.7a
Average	66.7 ± 7.4A	175 ± 20.7A	–	64.1 ± 18.7A	30.5 ± 2.5B	72.7 ± 7.7B	–	59.4 ± 17.7A
Background^b,d^	0.4–46	12–42	–	12–35	0.4–46	12–42	–	12–35

*Note*: The O‐horizon and A‐horizon data at both sites represent means of 24 observations. O‐horizon and A‐horizon data are based on six transects at four distances (3, 5, 10, and 30 m), resulting in 24 observations per horizon (mean of six observations per distance for each site). Earthworms’ data are means of three observations at National Capital Parks‐East (NACE) and six observations at Rock Creek Park (ROCR) for each distance. Distance means within a site and column followed by the same lowercase letter or site means in a column followed by the same uppercase letter are not significantly different at *p* < 0.05.

^a^ Hajar et al. (2014), ^b^Yesilonis et al. (2008); Richardsonet al. (2015b), ^c^Padmavathiamma and Li (2007), and ^d^Kabata‐Pendias (2000).

**FIGURE 5:**
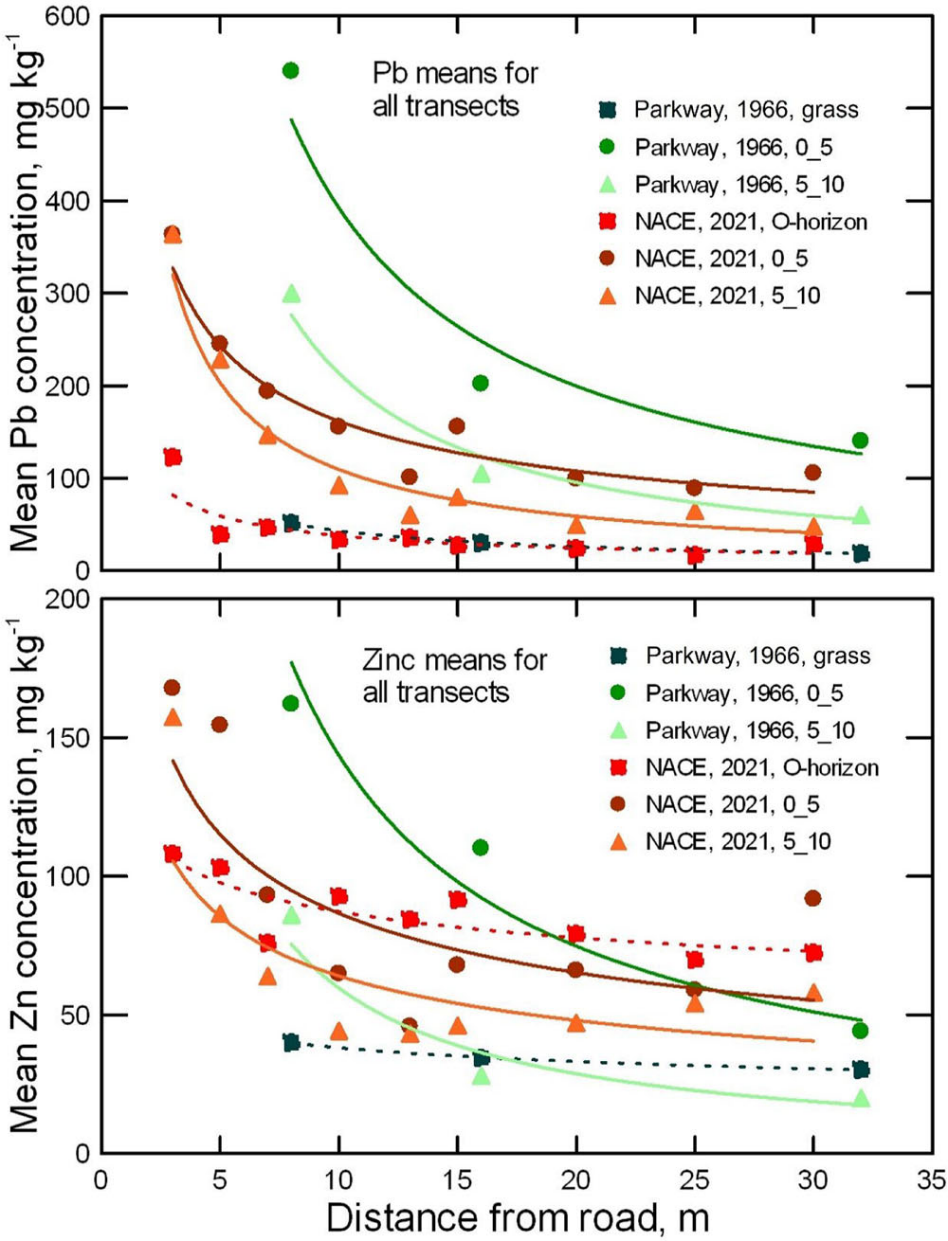
The Pb (upper panel) and Zn (lower panel) in mineral soil (solid lines) and organic material (dashed lines) at various distances from the edge of the highway in National Capital Parks‐East (NACE) for different soil depths, compared with historical data from the Washington‐Baltimore Parkway (Parkway) reported by Lagerwerff and Specht (1970).

Zinc concentrations in shallow soil layers exceeded natural parent material levels across all study sites (Figure 3), with typical central Maryland soils containing 73 mg kg^−1^ Zn and < 100 mg kg^−1^ in all major rocks in the region (Krauskopf & Bird, 1967; Rose, 1979; Yesilonis et al., 2008). In 1966, 8 m from the Baltimore Washington Parkway soil Zn concentrations at 0–10 cm averaged 96 mg kg^−1^ (Lagerwerff & Specht, 1970) while in the current study Zn in 0–10 cm (A‐horizon) soil at 7 m from the road was similar at 77.4 mg kg^−1^ (Figure 5) despite our expectation that Zn would be substantially higher in our 2020 samples due to the continued Zn emissions from tires, brakes, radiators, and oil (Dikwa et al., 2019). Tires typically contain 1.3%– 1.7% Zn (Ozaki et al., 2004) and16–90 mg Zn km^−1^ of travel are emitted from each tire (Baekken, 1992; Lee et al., 1997).

Early roadside heavy metal contamination studies such as Lagerwerff and Specht (1970) did not prioritize Cu measurement. However, in recent studies, for example (Szwalec et al., 2020), Cu is treated as an important contaminant from traffic. In our study, Cu concentrations ranged from 27.3 to 118 mg kg^−1^ in ROCR and NACE throughout 0 to 30‐cm soil depth (Figure 3). By comparison, the typical Cu concentration in mineral soil in Maryland is 12–42 mg kg^−1^ (Yesilonis et al., 2008).

At 0 to 2.5‐cm depth at 1 m from the road in NACE, Cu averaged 165 mg kg^−1^, the highest concentration in this study and four times greater than in the least contaminated soil. Therefore, our data support our hypothesis that Cu is accumulating in soil along heavily trafficked roads. Copper is a component of brake pads. However, in 2015 US vehicle manufacturers agreed to minimize Cu in brake pads (EPA, 2015). Copper in NACE was much higher than in ROCR (Figure 4), even for the samples least affected by traffic emissions (30 m from the road and 30 cm deep), suggesting that the soil parent material at NACE was inherently higher in Cu. However, the effect of proximity to the road was also much more pronounced at NACE (Figure 4), which could be related to the higher traffic volume and greater speed limit on Baltimore–Washington Parkway. Between 2014 and 2020 annual average daily traffic on Military road was 6911 compared to 103,000 on the Baltimore–Washington Parkway (Maryland Department of Transportation, 2020).

The Pb concentrations in all O‐horizon samples were much lower than in the mineral soil below, but much higher than typical Pb concentration in plants (Table 1) (Hajar et al., 2014; Marschner, 2011). O‐horizon Pb was significantly higher at NACE than at ROCR (48.9 ± 43.7 vs.16.8 ± 18.3 mg kg^−1^) but did not vary significantly with distance from the road (Figures 3 and 4). Additionally, taking into account the bulk density of the samples revealed the stocks of Pb in the mineral soil were high compared to O‐horizon (Table S2). These data are consistent with plant uptake and transport to foliage being drivers of O‐horizon composition. Not being a plant nutrient, Pb uptake and transport to foliage in trees is limited (Klaminder et al., 2006). In addition, leaf fall distribution is subject to wind, masking distance effects on Pb concentration. In another part of NACE during 1966, when leaded gasoline use was high, Pb concentrations in grass tissues ranged from 51.3 mg kg^−1^ at 8 m distance to 18.5 mg kg^−1^ at 32 m distance from the road (Lagerwerff & Specht, 1970), concentrations in the same range as those we found in O‐horizons in 2020 (Figure 5).

Previous research in the eastern United States (Richardson et al., 2015b) reported that Pb concentrations in mineral soil and O‐horizon were related (*r* = 0.56) and anthropogenic O‐horizon Pb appeared to move down and accumulate in underlying mineral soil layers (Kaste et al., 2003; Richardson et al., 2015a). In the current study, Pb in A‐horizon (0–10 cm) soil was only weakly correlated with Pb in O‐horizon material across both sites (Table S3), suggesting that because Pb is no longer being deposited from leaded fuel, the O‐horizon has little contribution to the elevated Pb level in the A‐horizon. The decrease in Pb concentration with soil depth (Figure 4), indicate that the elevated soil Pb likely resulted from historical aerial deposition rather than recent inputs from O‐horizon Pb.

The stock of Zn in the O‐horizon is very low compared to the A‐horizon (Table S3). Unlike for Pb, we expected Zn concentrations at all distances to exceed concentrations reported in grass tissue along the Baltimore–Washington Parkway in 1966 by Langerwerff and Specht (1970). While recognizing that comparing O‐horizon soil with grass tissue involves different materials and processes of Zn accumulation, the higher Zn concentration in recent O‐horizon samples suggests increased Zn deposition over time relative to the 1966 grass tissue measurements. In 1966, Zn concentration in grass tissue was 40.0 mg kg^−1^ at 8 m distance and 30.3 mg kg^−1^ at 32 m distance from the road in 1966 (Figure 5) (Lagerwerff & Specht, 1970). Our higher Zn concentrations may reflect continued emissions, increased traffic volume on the Baltimore–Washington Parkway (36,000 cars day^−1^ in 1970 [Gish & Christensen, 1973] to 103,000 cars day^−1^ in 2020 [Maryland Department of Transportation, 2020]), and continued biocycling.

Unlike Pb, Zn is an essential nutrient that plants readily take up and biocycle. The O‐horizon material is derived mainly from tree litterfall, but the concentrations of metals would be expected to increase during the decomposition process as carbon is lost due to respiration, but the metals are mainly retained in the material. We did not measure metal content of tree foliage, but normal Zn concentrations in oak (*Quercus* spp.) leaves have been reported as being between 14 and 28 mg kg^−1^ (Stojnić et al., 2019), and typical Zn in plants ranges from 10 to 150 mg kg^−1^ (Padmavathiamma & Li, 2007). Plant Zn toxicity can occur with tissue concentrations as low as 90–100 mg kg^−1^, but toxicity levels vary according to plant type and development stage (Baycu et al., 2006).

The typical range of Cu in most plants is 0.4–46 mg kg^−1^ (Kabata‐Pendias, 2000). Across all distances, O‐horizon Cu concentrations were generally approximately half those in the A‐horizon soil below, while the stock of Cu is much lower in the O‐horizon than in the 30 cm soil (Table S2). These findings suggest that O‐horizon Cu levels are influenced by current Cu deposition, while A‐horizon Cu may reflect the legacy of Cu accumulation. Kabala et al. (2014) reported a positive relationship between dissolved organic carbon and Cu concentration in litter. Copper is more readily mobilized from litter than Pb. However, leaching is not likely the dominant factor decreasing Cu in the O‐horizon (Richardson et al., 2015b). In northeastern US forest study, mean O‐horizon Cu was 7.9 mg kg^−1^ in 2011, which was much lower than 14.6 mg kg^−1^ in 1980 (Richardson et al., 2015a). According to that study, heavy metals such as Cu are diluted by cumulative increase in O‐horizon biomass over time.

### 3.2 Earthworm accumulation of Pb, Zn, and Cu

Earthworms tended to have higher Pb concentrations nearer the road, but this trend was significant only in NACE. Earthworms Pb concentration averaged from 32.4 at 30 m to 91.4 mg kg^−1^ at 3 m from the road in ROCR and from 23.5 to 186 mg kg^−1^, respectively, in NACE (Table 1). The normal range of Pb in various earthworm species in uncontaminated soil concentration ranges is 0.24–0.8 mg kg^−1^ (Richardson et al., 2015b). Overall average earthworm tissue Pb at NACE was 125 ± 204 mg kg^−1^, significantly higher than 58 ± 52 mg kg^−1^ at ROCR, following the pattern of the O‐horizon rather than the A‐horizon.

Earthworms have been shown to accumulate relatively high levels of metals from soils contaminated by various anthropogenic sources (Beyer et al., 1982; Martin, 2012; Wang et al., 2018). Earthworms ingest organic and mineral material, but their principal food is the microbial biomass associated with these soil materials (McInerney & Bolger, 2000). Two explanations exist for earthworm Pb levels. They may have bioaccumulated Pb from food, but it's unclear if their primary food comes from the O or A‐horizon. Alternatively, Pb exposure could be a combination of both horizons (Table 1). We did not identify earthworms by species, and it is possible that some were epigeic and active only in O‐horizons. Gish and Christensen (1973) represented that Lumbricus terrestris, Allolobophora chlorotica, Aporrectodea trapezoides, and Aporrectodea tuberculata are the most common earthworm species along the Baltimore–Washington Parkway in southern Maryland. Earthworm Pb was weakly correlated with O‐horizon Pb and A‐horizon Pb across the two sites (Table S3). Research in 22 New York sites showed that unlike most trace elements, Pb concentrations in earthworms were predicted by Pb soil concentrations. Bioaccumulation may be dependent on trace element bioavailability and mobility (Richardson, 2019). Trace elements occurring as salts are more bioavailable than those highly complexed with secondary minerals (Dai et al., 2004; Richardson et al., 2015a). Earthworms may ingest fresh litter with low trace element concentrations or mineral soil with high trace element concentrations (Chang et al., 2018). Other studies also observed that soil Pb concentration was linked with Pb accumulation in earthworms (Becquer et al., 2005; Dai et al., 2004; Little, 2011; Marinussen et al., 1997).

The Pb BSAF for earthworms versus A‐horizons 0.72 at NACE and 0.37 at ROCR (Table S3). A‐horizon Pb BSAF values exhibited no significant variation with distance from the road. The comparatively low Pb levels in earthworms compared to A‐horizon soil may be attributed to sorption and chelation reactions that restrict Pb mobility and bioavailability in the A‐horizon (Bolan et al., 2014; Rodríguez‐Seijo et al., 2017; Wang et al., 2018) or to epigeic feeding patterns. On mine‐tailing contaminated pasture soil, Dai et al. (2004) reported that Pb BSAF was < 1 for two species of earthworms (0.08–0.38 in *Aporrectodea caliginosa* and 0.04–0.13 in *Lumbricus rubellus*). Gish and Christensen (1973) observed that Pb in Maryland earthworms declined from 331 to 67.4 mg kg^−1^ from 3 to 49 m from the road (Gish & Christensen, 1973).

The average Zn concentration in earthworms ranged from 192 mg kg^−1^ to 592 mg kg^−1^; however, in uncontaminated soil, the range of Zn concentration spans from 152 to 330 mg kg^−1^ in various earthworm species (Richardson et al., 2015b). The Zn concentrations in mineral A‐horizon soil were significantly higher in ROCR than in NACE, but the two sites did not differ significantly in O‐horizon and earthworm Zn concentrations.

Zinc concentration in earthworms showed a non‐significant (*p* > 0.05) decreasing trend with distance from the road (Table 1). Interactions of transect direction with soil depth and distance from the road were significant for Zn at ROCR, but not at NACE (Table 2). For example, in NACE, A‐horizon Zn showed a clear decline with increasing distance from road to 10 m, with little further decline up to 30 m (Table 1; Figure 4). In contrast, at ROCR, where the transect directions were more perpendicular to the prevailing winds, A‐horizon Zn was generally higher than at NACE, but more variable with distance from the road. Zinc concentrations decreased significantly with mineral soil depth to 15 cm, especially within 7 m of the road (Figure 4) suggesting that elevated Zn, like Pb discussed earlier, came from aerial deposition of traffic emissions.

**TABLE 2:** Analysis of variance (ANOVA) summary table for each main and interaction effect resulting from factorial repeated measures ANOVA in two National Park forests for concentrations (mg kg^−1^) of Pb, Zn, and Cu in the O‐horizon and mineral soil samples to 30‐cm depth.

		F‐statistic		
Variables	*df*	Pb	Zn	Cu
**National Capital Parks‐East (NACE)**				
Depth	8	17.487***	15.283***	60.897***
Transect_direction	1	4.369*	1.141^ns^	35.559***
Distance	9	26.508***	49.31***	22.991***
Depth:Transect_direction	8	0.392^ns^	0.66^ns^	0.409^ns^
Depth:Distance	72	1.924***	2.15***	1.377*
Transect_direction:Distance	9	1.274^ns^	1.539^ns^	3.678***
Residuals	432	NA	NA	NA
**Rock Creek Park (ROCR)**				
Depth	8	38.5***	16.328***	44.586***
Transect_direction	1	3.306^ns^	0.469^ns^	0.274^ns^
Distance	9	26.371***	10.821***	11.891***
Depth:transect_direction	8	0.838^ns^	3.871***	0.331^ns^
Depth:distance	72	1.588**	1.339*	1.573**
Transect_direction:distance	9	6.37***	9.75***	1.499^ns^
Residuals	432	NA	NA	NA

*p < 0.05; **p < 0.01; ***p < 0.001. ns not significant.

We expected that earthworms would bioaccumulate Zn from the O‐horizon and A‐horizon soil. However, while Zn concentrations in earthworms were significantly correlated with Zn concentrations in the O‐horizon in NACE and with A‐horizon Zn in ROCR, earthworm Zn was not correlated with A‐horizon Zn in NACE or with O‐horizon Zn in ROCR (Table S3). Table 1 shows that earthworms in both NACE and ROCR were two to four times as concentrated in Zn as the O‐horizon and A‐horizon soil where they were collected. Previous research reported that earthworms have higher Zn concentrations than the O‐horizon and mineral soil (Gish & Christensen, 1973; Morgan & Morgan, 1988; Richardson et al., 2015a; Wang et al., 2018). Nannoni et al. (2014) explained that Zn is essential for earthworm growth, maturation, and reproduction, and may be required for metabolic activities. In our study, the Zn BSAF values for earthworms compared to A‐horizon Zn concentrations, averaged 3.85 at NACE and 1.69 at ROCR. The BSAF values based on either O‐horizon or A‐horizon Zn concentrations did not vary with distance from the road. These Zn BSAF values are in the same range (1.95–7.91) as reported for *Lumbricus rubellus* worms in mine‐tailing contaminated pasture soil (Dai et al., 2004).

The Cu concentrations in earthworms ranged from 116 mg kg^−1^ (at 3 m distance) to 36.9 mg kg^−1^ (at 30 m distance) in ROCR and from 132 mg kg^−1^ (at 5 m distance) to 37.2 mg kg^−1^ (at 30 m distance) in NACE, while the typical Pb concentration range in various earthworm species inhabiting uncontaminated soil is 12–35 mg kg^−1^ (Richardson et al., 2015b). These data showed the earthworm Cu followed the same pattern as O‐horizon and A‐horizon Cu (Table 1). ROCR had significantly lower average Cu concentrations in the A‐ and O‐horizons than NACE, although the two sites had very similar Cu concentrations in earthworm tissue (Table 1).

Earthworms Cu was not significantly correlated with O‐horizon or A‐horizon Cu in NACE or ROCR (Table S3). Table 1 shows that earthworms in both NACE and ROCR were two to four times as concentrated in Cu as the O‐horizon and A‐horizon soil where they were collected. Some studies have reported a positive correlation between Cu in soil and earthworms and concluded that earthworms bioaccumulate Cu (Morgan & Morgan, 1988; Nannoni et al., 2014), while other research did not observe this association (Marinussen et al., 1997; Richardson, 2019). In our study, the Cu BSAF values for earthworms relative to A‐horizon Cu concentrations were significantly lower in NACE (0.51) than in ROCR (1.1) (Table S3). We speculate that this difference could result from more epigeic earthworm species at one site than the other. BSAF values from O‐horizon or A‐horizon Cu concentrations remained constant with distance from the road. These observed Cu BSAF values align with the range (0.27–0.81) reported for *L. rubellus* and *A. caliginosa* worms in mine‐tailing contaminated pasture soil (Dai et al., 2004).

### 3.3 Influence of road distance and depth on Pb, Zn, and Cu

In the upper layers of mineral soil, Pb concentrations declined logarithmically with distance from the road (Figure S1). For example, for 0–2.5 cm soil at ROCR:

$$c=340-74.6\times \ln\left(x\right),R^{2}=0.30p\;<\;0.0001$$

with *c* the Pb concentration (mg kg^−1^) and *x* the distance from the road (m).

We expected lower Pb in soil, O‐horizon, and worms at 30 m from the road, as most Pb particles emitted from leaded gasoline combustion would settle near the road, with only fine particles carried by wind up to 30 m. In southern France, on a flat, treeless, windy grassland with a dry Mediterranean climate, during the use of leaded gasoline, Pb concentrations in the top 1–5 cm of roadside soil decreased with distance from traffic, as Pb‐containing particulates were transported by wind up to 500 m away (Piron‐Frenet et al., 1994). Their open site contrasted with ours, which was heavily forested, sloping, and in a humid temperate region. For Pb in ROCR there were significant effects for distance, depth, distance × direction, and soil depth × distance (Table 2). For Pb in NACE, all of the above‐mentioned effects were significant except for distance × transect direction (Table 2). Greater Pb accumulation on the southeast side at 3–15 m from the road suggests a relationship with the prevailing wind from the northwest (Figure 1, inset). At both sites, soil Pb concentrations decreased significantly (*p* < 0.001) with distance from the road (Figure 3). In 1966, in nearby locations along the B–W Parkway soil Pb at 0 to 10‐cm depth ranged from 300 to 540 mg kg^−1^ at 8 m from the road (Figure 5) (Lagerwerff & Specht, 1970). Despite the phase out of gasoline Pb beginning in 1975 (Newell & Rogers, 2003), in 2020 we still observed elevated soil Pb concentrations (210–1015 mg kg^−1^ at 1 m distance and 202 to 580 mg kg^−1^ at 7 m distance within the 0 to 10‐cm of soil depth (Figures 3 and 4).

At all locations along the sample transects in both parks, Pb concentrations generally declined with soil depth (from 0 to 30 cm), but below 10 cm the decline was not statistically significant (Figure 3). For example, in NACE, at 1 and 3 m from the road, respectively, mean mineral soil Pb concentrations declined from 204 to 369 mg kg^−1^ at 0–2.5 cm to only 178 and 55.2 mg kg^−1^, respectively, at 25–30 cm (Figure 3). We hypothesized this vertical gradient is due to Pb deposition on the soil surface and low mobility by leaching (Bezuglova et al., 2016; Impens et al., 1970). The decline in Pb concentrations with depth was less pronounced at greater distances. The difference in distribution between two sites is likely due to lower soil CEC, Ca, and OM content in NACE (Table S1). Vertical migration of heavy metals in soil can be influenced by soil texture, structure, CEC, clay content, SOM, and soil pH (e.g., Benhachem & Harrache, 2021; Bezuglova et al., 2016; Ran, 2018; Sterckeman et al., 2000).

The concentrations of Pb and Zn in the soil minerals and O‐horizon showed a similar pattern of increasing concentrations with road proximity at both sites (Table1; Figures 3 and 4). Deeper soil Zn concentrations were higher in ROCR compared to NACE, although Zn levels in shallow soil near the road were similar in both parks (Figure 4; Figure S2). Similar spatial trends in soil Zn have been reported in a range of other locations such as Nigeria, Lithuania, and Maryland (Dikwa et al., 2019; Grigalaviciene et al., 2005; Lagerwerff & Specht, 1970), with researchers concluding that long‐term heavy metal pollution from vehicles is the cause (Grigalaviciene et al., 2005). As for the A‐horizon, O‐horizon Zn concentrations also decreased with distance from the road, from 154 at 1 m to 66.8 mg kg^−1^at 30 m from the road, averages across both parks (Figure 3; Table 1). Most vehicle Zn contaminants are particles likely carried by wind and deposited on top of the soil, although vegetation can act as a biofilter between the air and the soil (Szwalec et al., 2020).

Copper concentration decreased significantly with soil depth from 0 to 30 cm, especially near the road (Figure 3, Figure S3; Tables 1 and 2). Also, the interaction effect between direction, distance, and depth was significant for Cu (Figure S4; Table 2). Copper concentrations showed a significant decrease with distance from the road in both parks (Table 1). The lowest Cu concentration was found 30 m from the road, while the highest Cu concentrations was near the road (Figure 3). Like A‐horizon Cu, O‐horizon Cu concentrations decreased from 70.8 mg kg^−1^ at 1 m to 65.6 mg kg^−1^ at 30 m from the road in ROCR. In NACE, the corresponding decrease in O‐horizon Cu was only from 36.1 to 30.9 mg kg^−1^ (Figure 3 and Table 1).

Compared to Pb and Zn, Cu concentrations were less variable and declined less dramatically with soil depth and distance from the road (Figure 4 and Table 1). Previous research reported that Cu decreased only slightly (from 11 to 8.0 mg kg^−1^) in surface mineral soil from the road edge to 140 m away (Szwalec et al., 2020). Larger Cu particles are mostly deposited near the road, whereas the smallest particles can stay suspended to greater distances (Masoudi et al., 2012). Generally, at all distances, Cu concentration declined from 0–2.5 cm through 30 cm of soil depth (Figure 4), which is related to low mobility of Cu due to bonding with soil minerals and organic matter. In ROCR, the Cu concentration within 3 m from the road was highest in the top 5 cm of soil, while in NACE, Cu was more evenly distributed throughout the soil profile, likely due to the lower soil CEC and OM in NACE, which facilitates movement of Cu (Figure S3; Table 1). Small amounts of dissolved and exchangeable Cu are likely in equilibrium with greater quantities of strongly adsorbed forms of Cu (McLaren & Crawford, 1973). Copper in soils has relatively low mobility as it is easily precipitated by sulfides, carbonates, and hydroxides and bound by organic matter (Minkina et al., 2014).

## 4 CONCLUSION

All three metals, Pb, Zn, and Cu, exhibited a distinct pattern of accumulation near the soil surface, and that pattern was most pronounced at locations nearest the road, thus confirming our hypothesis that emissions from vehicles contaminate roadside soils and forest ecosystems. Despite the phaseout of Pb in gasoline approximately 40 years before our study, this metal was still present in the mineral soil at levels similar to those reported from a study in 1966 at nearby sites. It appears that Pb did not cycle through the trees into the organic O horizon and did not accumulate in earthworm tissues. In contrast, Zn appears to be still accumulating and cycling through trees, into the organic O horizon and into earthworms.

Lead, emitted from vehicle fuel in volatile forms, contaminated upper soil layers farther from the road compared to Zn or Cu. The latter metals are less likely to travel long distances through densely forested systems via wind. At 30 m from the road, Pb at 0–10 cm was approximately 3–4 times more concentrated than at 25–30 cm. For Zn and Cu, there was no significant concentration difference between the shallowest and deepest layers at distances of 15 m or more from the roadway. Our data indicate that legacy Pb contamination remains persistent and is slowly moving down the soil profile and/or being buried by sediments. Zinc presents an ongoing contamination challenge as it is emitted from rubber tire wear. Copper exhibited the least ability to disperse from the roadway. Variations in Cu concentration within a single site were likely more influenced by inherent differences in the soil parent material than by the distance from the road.

## AUTHOR CONTRIBUTIONS

**Maryam Foroughi**: Data curation; formal analysis; investigation; software; validation; visualization; writing—original draft; writing—review and editing. **Raymond R. Weil**: Conceptualization; formal analysis; funding acquisition; investigation; methodology; project administration; software; supervision; validation; visualization; writing—review and editing.

## CONFLICT OF INTEREST STATEMENT

The authors declare no conflicts of interest.

## Supporting information



Supplemental Material

Supplemental Material

## References

[jeq220642-bib-0002] Abdelhafez, A. A. , Abbas, M. H. , & Attia, T. (2015). Environmental monitoring of heavy‐metals status and human health risk assessment in the soil of Sahl El‐Hessania area, Egypt. Polish Journal of Environmental Studies, 24(2), 459–467.

[jeq220642-bib-0003] Alloway, B. J. (2012). Heavy metals in soils: Trace metals and metalloids in soils and their bioavailability (Vol. 22). Springer Science & Business Media.

[jeq220642-bib-0004] Ardestani, M. M. , Giska, I. , & van Gestel, C. A. (2019). The effect of the earthworm Lumbricus rubellus on the bioavailability of cadmium and lead to the springtail Folsomia candida in metal‐polluted field soils. Environmental Science and Pollution Research, 26(27), 27816–27822. 10.1007/s11356-019-05969-3 31342354

[jeq220642-bib-0005] Awofolu, O. (2004). Impact of automobile exhaust on levels of lead in a commercial food from bus terminals. Journal of Applied Sciences and Environmental Management, 8(1), 23–27.

[jeq220642-bib-0006] Bååth, E. (1989). Effects of heavy metals in soil on microbial processes and populations (a review). Water, Air, and Soil Pollution, 47(3), 335–379. 10.1007/BF00279331

[jeq220642-bib-0007] Baekken, T. (1993). Environmental effects of asphalt and tyre wear by road traffic. In Nordic Seminar and Work Reports (p. 628). Nordic Co‐Operation.

[jeq220642-bib-0008] Baycu, G. , Tolunay, D. , Özden, H. , & Günebakan, S. (2006). Ecophysiological and seasonal variations in Cd, Pb, Zn, and Ni concentrations in the leaves of urban deciduous trees in Istanbul. Environmental Pollution, 143(3), 545–554. 10.1016/j.envpol.2005.10.050 16480798

[jeq220642-bib-0009] Becquer, T. , Dai, J. , Quantin, C. , & Lavelle, P. (2005). Sources of bioavailable trace metals for earthworms from a Zn‐, Pb‐ and Cd‐contaminated soil. Soil Biology and Biochemistry, 37(8), 1564–1568. 10.1016/j.soilbio.2005.01.007

[jeq220642-bib-0010] Benhachem, F. Z. , & Harrache, D. (2021). Chemical speciation and potential mobility of heavy metals in forest soil near road traffic in Hafir, Algeria. Journal of Health Pollution, 11(30), 210614. 34268001 10.5696/2156-9614-11.30.210614 PMC8276720

[jeq220642-bib-0011] Beyer, W. N. , Chaney, R. L. , & Mulhern, B. M. (1982). Heavy metal concentrations in earthworms from soil amended with sewage sludge. Journal of Environmental Quality, 11(3), 381–385. 10.2134/jeq1982.00472425001100030012x

[jeq220642-bib-0012] Bezuglova, O. S. , Gorbov, S. N. , Tischenko, S. A. , Aleksikova, A. S. , Tagiverdiev, S. S. , Sherstnev, A. K. , & Dubinina, M. N. (2016). Accumulation and migration of heavy metals in soils of the Rostov region, south of Russia. Journal of Soils and Sediments, 16(4), 1203–1213. 10.1007/s11368-015-1165-8

[jeq220642-bib-0013] Birch, G. , & Scollen, A. (2003). Heavy metals in road dust, gully pots and parkland soils in a highly urbanised sub‐catchment of Port Jackson, Australia. Soil Research, 41(7), 1329–1342.

[jeq220642-bib-0014] Bolan, N. , Kunhikrishnan, A. , Thangarajan, R. , Kumpiene, J. , Park, J. , Makino, T. , Kirkham, M. B. , & Scheckel, K. (2014). Remediation of heavy metal(loid)s contaminated soils—To mobilize or to immobilize? Journal of Hazardous Materials, 266, 141–166. 10.1016/j.jhazmat.2013.12.018 24394669

[jeq220642-bib-0015] Chang, C.‐H. , Johnston, M. R. , Görres, J. H. , Dávalos, A. , McHugh, D. , & Szlavecz, K. (2018). Co‐invasion of three Asian earthworms. Metaphire hilgendorfi, Amynthas agrestis and Amynthas tokioensis in the USA. Biological Invasions, 20(4), 843–848.

[jeq220642-bib-0016] Chrzan, A. (2013). Content of heavy metals in soil and in pine bark. Proceedings of ECOpole, 7, 547–552. 10.2429/proc.2013.7(2)072

[jeq220642-bib-0017] Dai, J. , Becquer, T. , Rouiller, J. H. , Reversat, G. , Bernhard‐Reversat, F. , Nahmani, J. , & Lavelle, P. (2004). Heavy metal accumulation by two earthworm species and its relationship to total and DTPA‐extractable metals in soils. Soil Biology and Biochemistry, 36(1), 91–98. 10.1016/j.soilbio.2003.09.001

[jeq220642-bib-0018] Dikwa, M. K. , Akan, J. C. , & Adamu, A. (2019). Determination of some heavy metals in roadside soils from some major roads in Maiduguri, Borno State, Nigeria. Nuclear Science, 4(3), 27–33. 10.11648/j.ns.20190403.11

[jeq220642-bib-0019] Dragicevic, I. , Eich‐Greatorex, S. , Sogn, T. A. , Linjordet, R. , & Krogstad, T. (2017). Fate of copper, nickel and zinc after biogas digestate application to three different soil types. Environmental Science and Pollution Research, 24(14), 13095–13106. 10.1007/s11356-017-8886-8 28382449

[jeq220642-bib-0020] Einberger, S. (2014). A history of Rock Creek Park: Wilderness & Washington, DC. Arcadia Publishing.

[jeq220642-bib-0021] Ekenler, M. , & Tabatabai, M. (2002). Effects of trace elements on β‐glucosaminidase activity in soils. Soil Biology and Biochemistry, 34(11), 1829–1832. 10.1016/S0038-0717(02)00167-0

[jeq220642-bib-0022] Fox, C. A. , Miller, J. J. , Joschko, M. , Drury, C. F. , & Reynolds, W. D. (2017). Earthworm population dynamics as a consequence of long‐term and recently imposed tillage in a clay loam soil. Canadian Journal of Soil Science, 97(4), 561–579.

[jeq220642-bib-0023] Friedland, A. J. , Johnson, A. H. , & Siccama, T. G. (1986). Coniferous litter decomposition on Camels Hump, Vermont: A review. Canadian Journal of Botany, 64(7), 1349–1354. 10.1139/b86-186

[jeq220642-bib-0024] Gee, G. , & Or, D. (2002). Particle‐size analysis. In J. H. Dane & G. C. Topp (Eds.), Methods of soil analysis: Part 4 physical methods (Vol. 4, pp. 255–293). ASA, CSSA, SSSA. 10.2136/sssabookser5.4.c12

[jeq220642-bib-0025] Gish, C. D. , & Christensen, R. E. (1973). Cadmium, nickel, lead, and zinc in earthworms from roadside soil. Environmental Science & Technology, 7(11), 1060–1062. 22263952 10.1021/es60083a011

[jeq220642-bib-0026] Grigalaviciene, I. , Rutkoviene, V. , & Marozas, V. (2005). The accumulation of heavy metals Pb, Cu and Cd at roadside forest soil. Polish Journal of Environmental Studies, 14(1), 109–115.

[jeq220642-bib-0027] Hajar, E. W. I. , Sulaiman, A. Z. B. , & Sakinah, A. M. (2014). Assessment of heavy metals tolerance in leaves, stems and flowers of *Stevia rebaudiana* plant. Procedia Environmental Sciences, 20, 386–393. 10.1016/j.proenv.2014.03.049

[jeq220642-bib-0028] Hansmann, W. , & Köppel, V. (2000). Lead‐isotopes as tracers of pollutants in soils. Chemical Geology, 171(1–2), 123–144. 10.1016/S0009-2541(00)00230-8

[jeq220642-bib-0029] Impens, I. , Lemeur, R. , & Moermans, R. (1970). Spatial and temporal variation of net radiation in crop canopies. Agricultural Meteorology, 7, 335–337. 10.1016/0002-1571(70)90028-2

[jeq220642-bib-0030] Johnson, A. , & Richter, S. (2010). Organic‐horizon lead, copper, and zinc contents of mid‐Atlantic forest soils 1978–2004. Soil Science Society of America Journal, 74(3), 1001–1009. 10.2136/sssaj2008.0337

[jeq220642-bib-0031] Kabala, C. , Karczewska, A. , & Medynska‐Juraszek, A. (2014). Variability and relationships between Pb, Cu, and Zn concentrations in soil solutions and forest floor leachates at heavily polluted sites. Journal of Plant Nutrition and Soil Science, 177(4), 573–584. 10.1002/jpln.201400018

[jeq220642-bib-0032] Kabata‐Pendias, A. (2000). Trace elements in soils and plants. CRC Press.

[jeq220642-bib-0033] Kassambara, A. , & Kassambara, M. A. (2020). *Package ‘ggpubr’* (R package version 0.1.6) [Computer software]. CRAN.

[jeq220642-bib-0034] Kaste, J. M. , Friedland, A. J. , & Stürup, S. (2003). Using stable and radioactive isotopes to trace atmospherically deposited Pb in montane forest soils. Environmental Science & Technology, 37(16), 3560–3567. 12953866 10.1021/es026372k

[jeq220642-bib-0035] Klaminder, J. , Bindler, R. , Emteryd, O. , Appleby, P. , & Grip, H. (2006). Estimating the mean residence time of lead in the organic horizon of boreal forest soils using 210‐lead, stable lead and a soil chronosequence. Biogeochemistry, 78(1), 31–49. 10.1007/s10533-005-2230-y

[jeq220642-bib-0036] Kovarik, W. (2005). Ethyl‐leaded gasoline: How a classic occupational disease became an international public health disaster. International Journal of Occupational and Environmental Health, 11(4), 384–397. 10.1179/oeh.2005.11.4.384 16350473

[jeq220642-bib-0037] Krauskopf, K. B. , & Bird, D. K. (1967). Introduction to geochemistry (Vol. 721). McGraw‐Hill.

[jeq220642-bib-0038] Lagerwerff, J. V. , & Specht, A. (1970). Contamination of roadside soil and vegetation with cadmium, nickel, lead, and zinc. Environmental Science & Technology, 4(7), 583–586.

[jeq220642-bib-0039] Lee, P.‐K. , Touray, J.‐C. , Baillif, P. , & Ildefonse, J.‐P. (1997). Heavy metal contamination of settling particles in a retention pond along the A‐71 motorway in Sologne, France. Science of the Total Environment, 201(1), 1–15. 9232021 10.1016/s0048-9697(97)84048-x

[jeq220642-bib-0040] Little, B. J. (2011). *Earthworm uptake and sequestration of lead in a terrestrial environment* [Master's thesis, Ohio State University].

[jeq220642-bib-0041] Lone, M. I. , He, Z.‐l. , Stoffella, P. J. , & Yang, X.‐e (2008). Phytoremediation of heavy metal polluted soils and water: Progresses and perspectives. Journal of Zhejiang University Science B, 9(3), 210–220. 10.1631/jzus.B0710633 18357623 PMC2266886

[jeq220642-bib-0042] Magdoff, F. R. , Tabatabai, M. A. , & Hanlon, E. A. (1996). Soil organic matter: Analysis and interpretation. ASA, CSSA, SSSA.

[jeq220642-bib-0043] Mälkönen, E. , Derome, J. , Fritze, H. , Helmisaari, H.‐S. , Kukkola, M. , Saarsalmi, A. , & Salemaa, M. (1999). Compensatory fertilization of Scots pine stands polluted by heavy metals. Nutrient Cycling in Agroecosystems, 55(3), 239–268.

[jeq220642-bib-0044] Marinussen, M. P. , van der Zee, S. E. , & de Haan, F. A. (1997). Cu accumulation in the earthworm *Dendrobaena veneta* in a heavy metal (Cu, Pb, Zn) contaminated site compared to Cu accumulation in laboratory experiments. Environmental Pollution, 96(2), 227–233. 10.1016/S0269-7491(97)00017-1 15093422

[jeq220642-bib-0045] Marschner, H. (2011). Marschner's mineral nutrition of higher plants. Academic Press.

[jeq220642-bib-0046] Martin, M. (2012). Biological monitoring of heavy metal pollution: Land and air. Springer Science & Business Media.

[jeq220642-bib-0047] Maryland Department of Transportation . (2020). *AADT'S of stations for the years* 2014–2020. Maryland State Highway Administration. https://www.roads.maryland.gov/oppen/station_history.pdf

[jeq220642-bib-0048] Masoudi, S. N. , Sepanlou, M. G. , & Bahmanya, M. (2012). Distribution of lead, cadmium, copper and zinc in roadside soil of Sari‐Ghaemshahr road, Iran. African Journal of Agricultural Research, 7(2), 198–204.

[jeq220642-bib-0049] McInerney, M. , & Bolger, T. (2000). Decomposition of *Quercus petraea* litter: Influence of burial, comminution and earthworms. Soil Biology and Biochemistry, 32(14), 1989–2000.

[jeq220642-bib-0050] McLaren, R. , & Crawford, D. (1973). Studies on soil copper I. The fractionation of copper in soils. Journal of Soil Science, 24(2), 172–181. 10.1111/j.1365-2389.1973.tb00753.x

[jeq220642-bib-0051] Mehlich, A. (1978). New extractant for soil test evaluation of phosphorus, potassium, magnesium, calcium, sodium, manganese and zinc. Communications in Soil Science and Plant Analysis, 9(6), 477–492. 10.1080/00103627809366824

[jeq220642-bib-0052] Minkina, T. , Mandzhieva, S. , Motusova, G. , Burachevskaya, M. , Nazarenko, O. , Sushkova, S. , & Kizilkaya, R. (2014). Heavy metal compounds in a soil of technogenic zone as indicate of its ecological state. Eurasian Journal of Soil Science, 3(2), 144–151.

[jeq220642-bib-0053] Möller, A. , Müller, H. , Abdullah, A. , Abdelgawad, G. , & Utermann, J. (2005). Urban soil pollution in Damascus, Syria: Concentrations and patterns of heavy metals in the soils of the Damascus Ghouta. Geoderma, 124(1–2), 63–71. 10.1016/j.geoderma.2004.04.003

[jeq220642-bib-0054] Morgan, J. , & Morgan, A. (1988). Earthworms as biological monitors of cadmium, copper, lead and zinc in metalliferous soils. Environmental Pollution, 54(2), 123–138. 10.1016/0269-7491(88)90142-X 15092529

[jeq220642-bib-0055] Nannoni, F. , Rossi, S. , & Protano, G. (2014). Soil properties and metal accumulation by earthworms in the Siena urban area (Italy). Applied Soil Ecology, 77, 9–17. 10.1016/j.apsoil.2014.01.004

[jeq220642-bib-0056] Newell, R. G. , & Rogers, K. (2003). The US experience with the phasedown of lead in gasoline. Resources for the Future.

[jeq220642-bib-0057] Nriagu, J. (1978). The biogeochemistry of lead. Elsevier.

[jeq220642-bib-0058] Ozaki, H. , Watanabe, I. , & Kuno, K. (2004). Investigation of the heavy metal sources in relation to automobiles. Water, Air, and Soil Pollution, 157(1), 209–223. 10.1023/B:WATE.0000038897.63818.f7

[jeq220642-bib-0059] Padmavathiamma, P. K. , & Li, L. Y. (2007). Phytoremediation technology: Hyper‐accumulation metals in plants. Water, Air, and Soil Pollution, 184(1), 105–126. 10.1007/s11270-007-9401-5

[jeq220642-bib-0060] Peech, M. (1965). Methods of soil analysis. Chemical and microbiological properties. American Society of Agronomy Madison.

[jeq220642-bib-0061] Piron‐Frenet, M. , Bureau, F. , & Pineau, A. (1994). Lead accumulation in surface roadside soil: Its relationship to traffic density and meteorological parameters. Science of the Total Environment, 144(1–3), 297–304. 10.1016/0048-9697(94)90449-9

[jeq220642-bib-0062] R Core Team . (2020). R: A language and environment for statistical computing. R Foundation for Statistical Computing. https://www.R‐project.org/

[jeq220642-bib-0063] Ran, D. (2018). Applied research in heavy metals pollution in expressway roadside soil in China. IOP Conference Series: Earth and Environmental Science, 170, 032137.

[jeq220642-bib-0064] Richardson, J. (2019). Trace elements in surface soils and *megascolecidae* earthworms in urban forests within four land‐uses around Poughkeepsie, New York, USA. Bulletin of Environmental Contamination and Toxicology, 103(3), 385–390. 10.1007/s00128-019-02669-z 31256200

[jeq220642-bib-0065] Richardson, J. , Donaldson, E. , Kaste, J. , & Friedland, A. (2015a). Forest floor lead, copper and zinc concentrations across the northeastern United States: Synthesizing spatial and temporal responses. Science of the Total Environment, 505, 851–859. 10.1016/j.scitotenv.2014.10.023 25461088

[jeq220642-bib-0066] Richardson, J. , Görres, J. , Jackson, B. , & Friedland, A. (2015b). Trace metals and metalloids in forest soils and exotic earthworms in northern New England, USA. Soil Biology and Biochemistry, 85, 190–198. 25883392 10.1016/j.soilbio.2015.03.001 PMC4395857

[jeq220642-bib-0067] Rodríguez‐Seijo, A. , Cachada, A. , Gavina, A. , Duarte, A. , Vega, F. , Andrade, M. , & Pereira, R. (2017). Lead and PAHs contamination of an old shooting range: A case study with a holistic approach. Science of the Total Environment, 575, 367–377. 10.1016/j.scitotenv.2016.10.018 27744202

[jeq220642-bib-0068] Rose, A. (1979). Geochemistry in mineral exploration. Academic Press.

[jeq220642-bib-0069] Ryu, C. (2019). *dlookr: Tools for data diagnosis, exploration, t*ransformation. CRAN.

[jeq220642-bib-0070] Salemaa, M. , & Uotila, T. (2001). Seed bank composition and seedling survivalin forest soil polluted with heavy metals. Basic and Applied Ecology, 2(3), 251–263. 10.1078/1439-1791-00055

[jeq220642-bib-0071] Schlesinger, W. H. , Reiners, W. A. , & Knopman, D. S. (1974). Heavy metal concentrations and deposition in bulk precipitation in montane ecosystems of New Hampshire, USA. Environmental Pollution, 6(1), 39–47.

[jeq220642-bib-0072] Schulte, E. , & Hopkins, B. (1996). Estimation of soil organic matter by weight loss‐on‐ignition. In F. R. Magdoff , M. A. Tabatabai , & E. A. Hanlon Jr. (Eds.), Soil organic matter: Analysis and interpretation (Vol. 46, pp. 21–31). ASA, CSSA, SSSA.

[jeq220642-bib-0073] Shoemaker, L. P. (1909). Historic rock creek. In Records of the Columbia *h*istorical *s*ociety, Washington, DC (Vol. 12, pp. 38–52). DC History Center.

[jeq220642-bib-0074] Siccama, T. G. , & Smith, W. H. (1978). Lead accumulation in a northern hardwood forest. Environmental Science & Technology, 12(5), 593–594.

[jeq220642-bib-0075] Sterckeman, T. , Douay, F. , Proix, N. , & Fourrier, H. (2000). Vertical distribution of Cd, Pb and Zn in soils near smelters in the North of France. Environmental Pollution, 107(3), 377–389. 10.1016/S0269-7491(99)00165-7 15092984

[jeq220642-bib-0076] Stojnić, S. , Kebert, M. , Drekić, M. , Galić, Z. , Kesić, L. , Tepavac, A. , & Orlović, S. (2019). Heavy metals content in foliar litter and branches of *Quercus petraea* (Matt.) Liebl. and *Quercus robur* L. observed at two ICP forests monitoring plots. South‐East European Forestry: SEEFOR, 10(2), 151–157. 10.15177/seefor.19-11

[jeq220642-bib-0077] Sumner, M. , & Miller, W. (1996). *Cation exchange capacity and exchange coefficients* . In D. L. Sparks , A. L. Page , P. A. Helmke , R. H. Loeppert , P. N. Soltanpour , M. A. Tabatabai , C. T. Johnston , & M. E. Sumner (Eds.), Methods of soil analysis part 3—Chemical methods (methodsofsoilan3) (pp. 1201–1229). ASA, CSSA, SSSA. 10.2136/sssabookser5.3.c40

[jeq220642-bib-0078] SYSTAT . (2022). *Statistical analysis and graphics software* (SYSTAT 13.2). Grafiti. https://systatsoftware.com/systat/

[jeq220642-bib-0079] Szwalec, A. , Mundała, P. , Kędzior, R. , & Pawlik, J. (2020). Monitoring and assessment of cadmium, lead, zinc and copper concentrations in arable roadside soils in terms of different traffic conditions. Environmental Monitoring and Assessment, 192(3), Article 155. 10.1007/s10661-020-8120-x 32006114 PMC6994438

[jeq220642-bib-0080] Trojan, M. , & Linden, D. (1994). Tillage, residue, and rainfall effects on movement of an organic tracer in earthworm‐affected soils. Soil Science Society of America Journal, 58(5), 1489–1494. 10.2136/sssaj1994.03615995005800050031x

[jeq220642-bib-0081] Trombulak, S. C. , & Frissell, C. A. (2000). Review of ecological effects of roads on terrestrial and aquatic communities. Conservation Biology, 14(1), 18–30. 10.1046/j.1523-1739.2000.99084.x

[jeq220642-bib-0082] Turer, D. , Maynard, J. B. , & Sansalone, J. J. (2001). Heavy metal contamination in soils of urban highways comparison between runoff and soil concentrations at Cincinnati, Ohio. Water, Air, and Soil Pollution, 132(3), 293–314. 10.1023/A:1013290130089

[jeq220642-bib-0083] Turner, R. , Johnson, A. , & Wang, D. (1985). Biogeochemistry of aluminum in McDonalds Branch watershed, New Jersey pine barrens. Journal of Environmental Quality (314–323).

[jeq220642-bib-0084] Tyler, G. (1976). Heavy metal pollution, phosphatase activity, and mineralization of organic phosphorus in forest soils. Soil Biology and Biochemistry, 8(4), 327–332. 10.1016/0038-0717(76)90065-1

[jeq220642-bib-0085] Tyler, G. , Påhlsson, A.‐M. B. , Bengtsson, G. , Bååth, E. , & Tranvik, L. (1989). Heavy‐metal ecology of terrestrial plants, microorganisms and invertebrates. Water, Air, and Soil Pollution, 47(3), 189–215. 10.1007/BF00279327

[jeq220642-bib-0086] US EPA . (2015). Copper‐free brake initiative . https://www.epa.gov/npdes/copper‐free‐brake‐initiative

[jeq220642-bib-0087] USDA NRCS . (2019). Web *s*oil *survey* . https://websoilsurvey.nrcs.usda.gov/app/

[jeq220642-bib-0088] Walsh, B. M. , Campbell, J. P. , Costanzo, S. D. , Dennison, W. C. , Lehman, M. , Milton, M. , Nortrup, M. , & Syphax, S. (2016). National Capital Parks‐East natural resource condition assessment . National Capital Region Parks.

[jeq220642-bib-0089] Wang, K. , Qiao, Y. , Zhang, H. , Yue, S. , Li, H. , Ji, X. , & Liu, L. (2018). Bioaccumulation of heavy metals in earthworms from field contaminated soil in a subtropical area of China. Ecotoxicology and Environmental Safety, 148, 876–883. 10.1016/j.ecoenv.2017.11.058 29605664

[jeq220642-bib-0090] Wang, Y. , Han, W. , Wang, X. , Chen, H. , Zhu, F. , Wang, X. , & Lei, C. (2017). Speciation of heavy metals and bacteria in cow dung after vermicomposting by the earthworm, *Eisenia fetida* . Bioresource Technology, 245, 411–418. 10.1016/j.biortech.2017.08.118 28898838

[jeq220642-bib-0091] Ward, N. , Brooks, R. , & Reeves, R. (1974). Effect of lead from motor‐vehicle exhausts on trees along a major thoroughfare in Palmerston North, New Zealand. Environmental Pollution, 6(2), 149–158. 10.1016/0013-9327(74)900317

[jeq220642-bib-0092] Ward, N. , Reeves, R. , & Brooks, R. (1975). Lead in soil and vegetation along a New Zealand state highway with low traffic volume. Environmental Pollution, 9(4), 243–251.

[jeq220642-bib-0093] Wheeler, G. , & Rolfe, G. (1979). The relationship between daily traffic volume and the distribution of lead in roadside soil and vegetation. Environmental Pollution, 18(4), 265–274.

[jeq220642-bib-0094] Wickham, H. (2016). ggplot2: Elegant graphics for data analysis. Springer.

[jeq220642-bib-0095] Wong, C. S. , Li, X. , & Thornton, I. (2006). Urban environmental geochemistry of trace metals. Environmental Pollution, 142(1), 1–16. 10.1016/j.envpol.2005.09.004 16297517

[jeq220642-bib-0096] Yesilonis, I. , Pouyat, R. , & Neerchal, N. (2008). Spatial distribution of metals in soils in Baltimore, Maryland: Role of native parent material, proximity to major roads, housing age and screening guidelines. Environmental Pollution, 156(3), 723–731. 10.1016/j.envpol.2008.06.010 18656291

[jeq220642-bib-0097] Zereini, F. , Alt, F. , Messerschmidt, J. , Wiseman, C. , Feldmann, I. , von Bohlen, A. , MÜller, J. , Liebl, K. , & PÜttmann, W. (2005). Concentration and distribution of heavy metals in urban airborne particulate matter in Frankfurt am Main. Environmental Science & Technology, 39(9), 2983–2989. 10.1021/es040040t 15926542

