# Supplementary material for: Soil lead, zinc, and copper in two urban forests as influenced by highway proximity
Source: J Environ Qual. 2024 Oct 21;54(1):275–88. doi: 10.1002/jeq2.20642 (PMC11718127; doi:10.1002/jeq2.20642)
Supplement: Supplementary file 1 — Supplemental Material [file JEQ2-54-275-s001.docx]

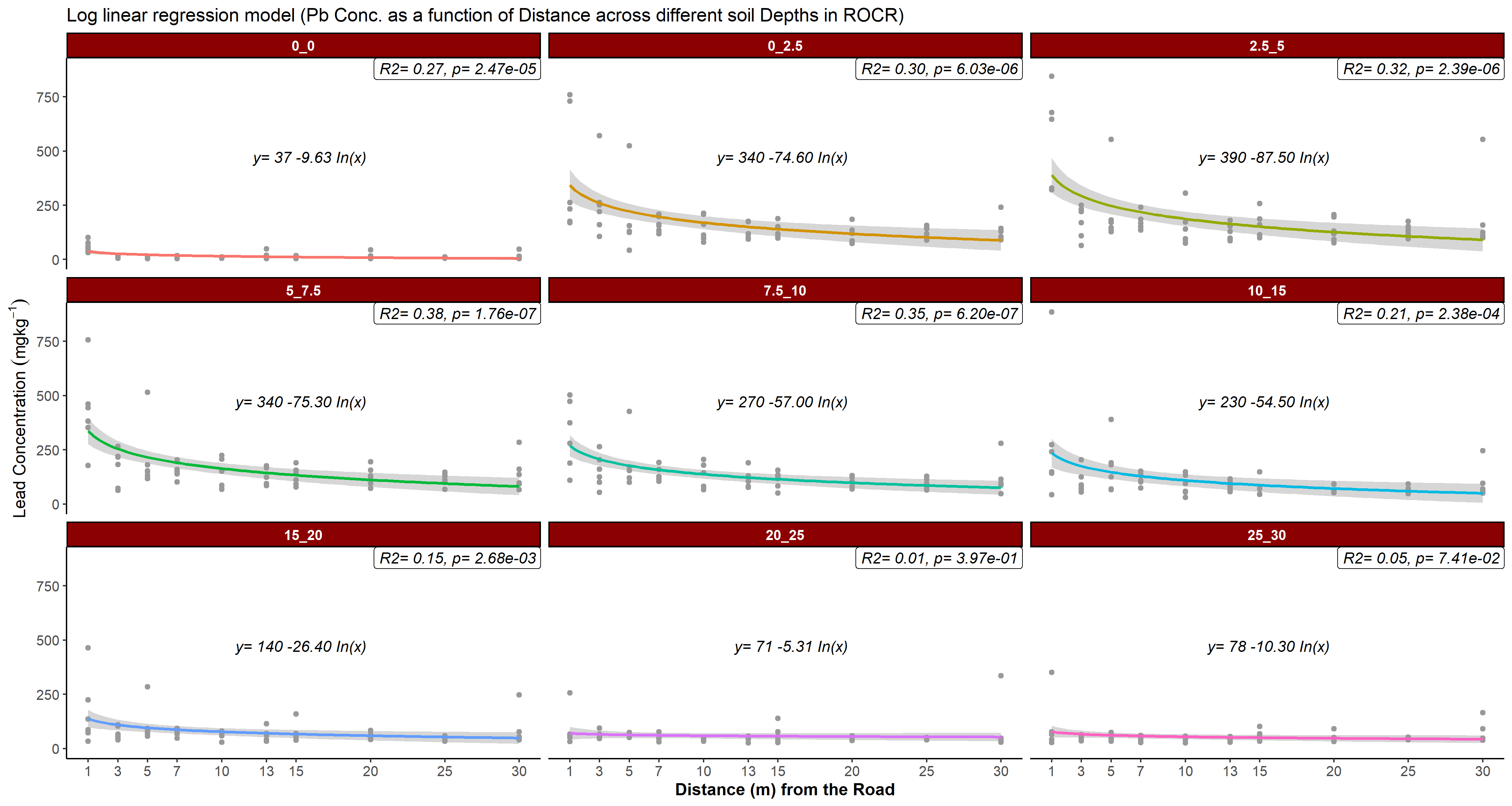


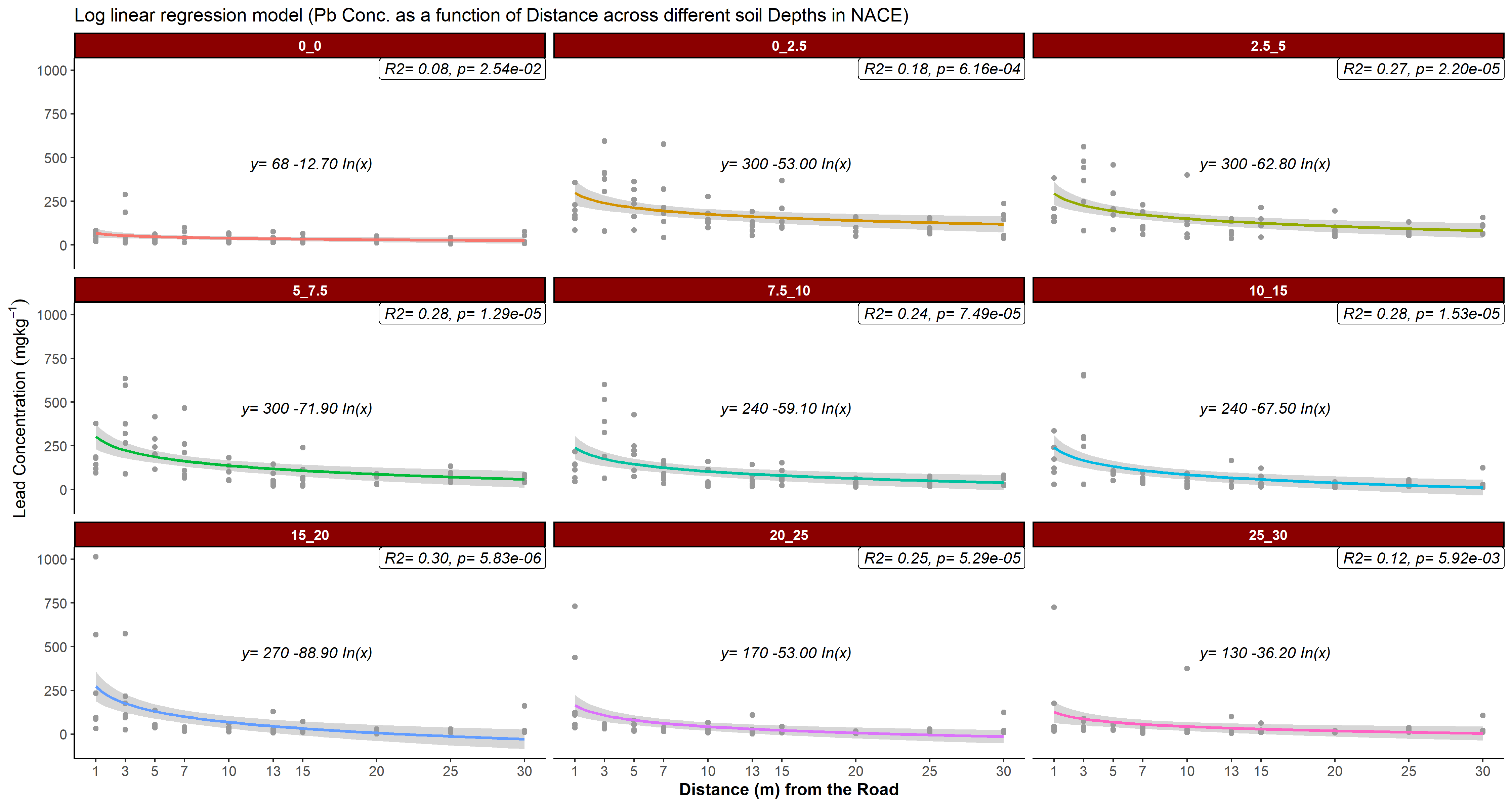


**Supplementary Figure 1.** Log linear regression model of between Pb concentration and road distance for each soil depth in Rock Creek National Park (ROCR) and NACE (National Capital Parks-East).


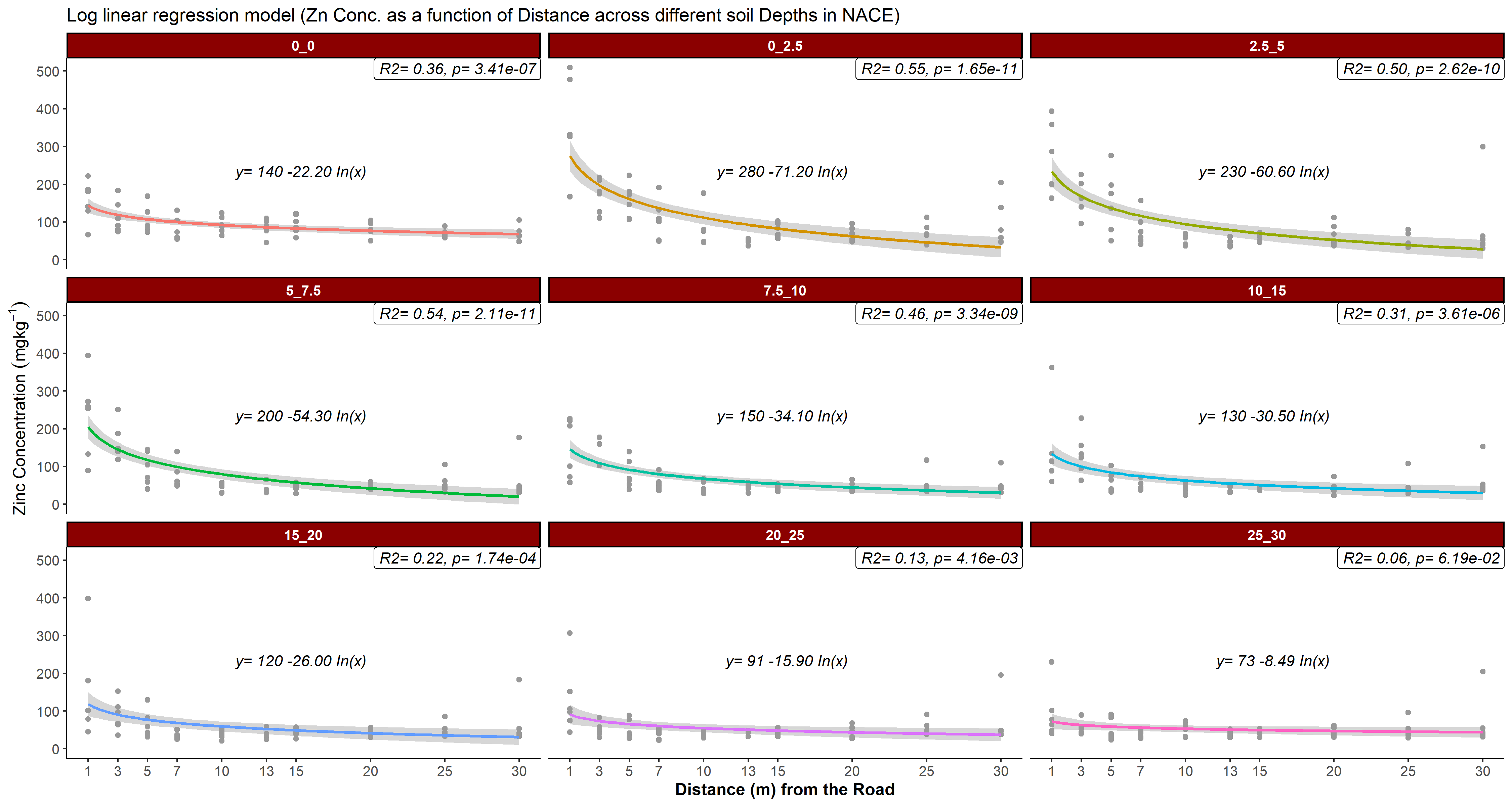

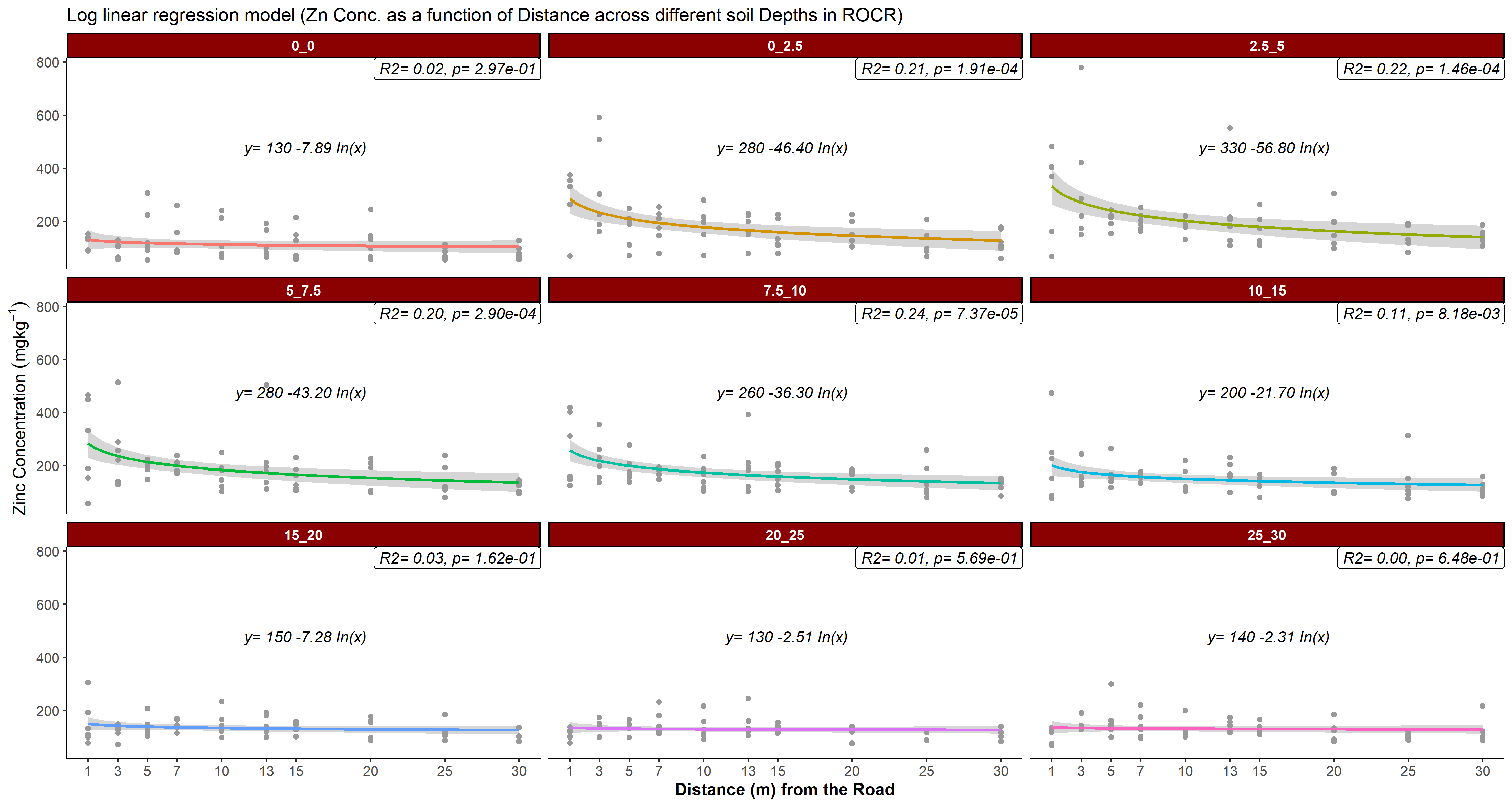


**Supplementary Figure 2.** Log linear regression model of between Zn concentration and road distance for each soil depth in Rock Creek National Park (ROCR) NACE (National Capital Parks-East).


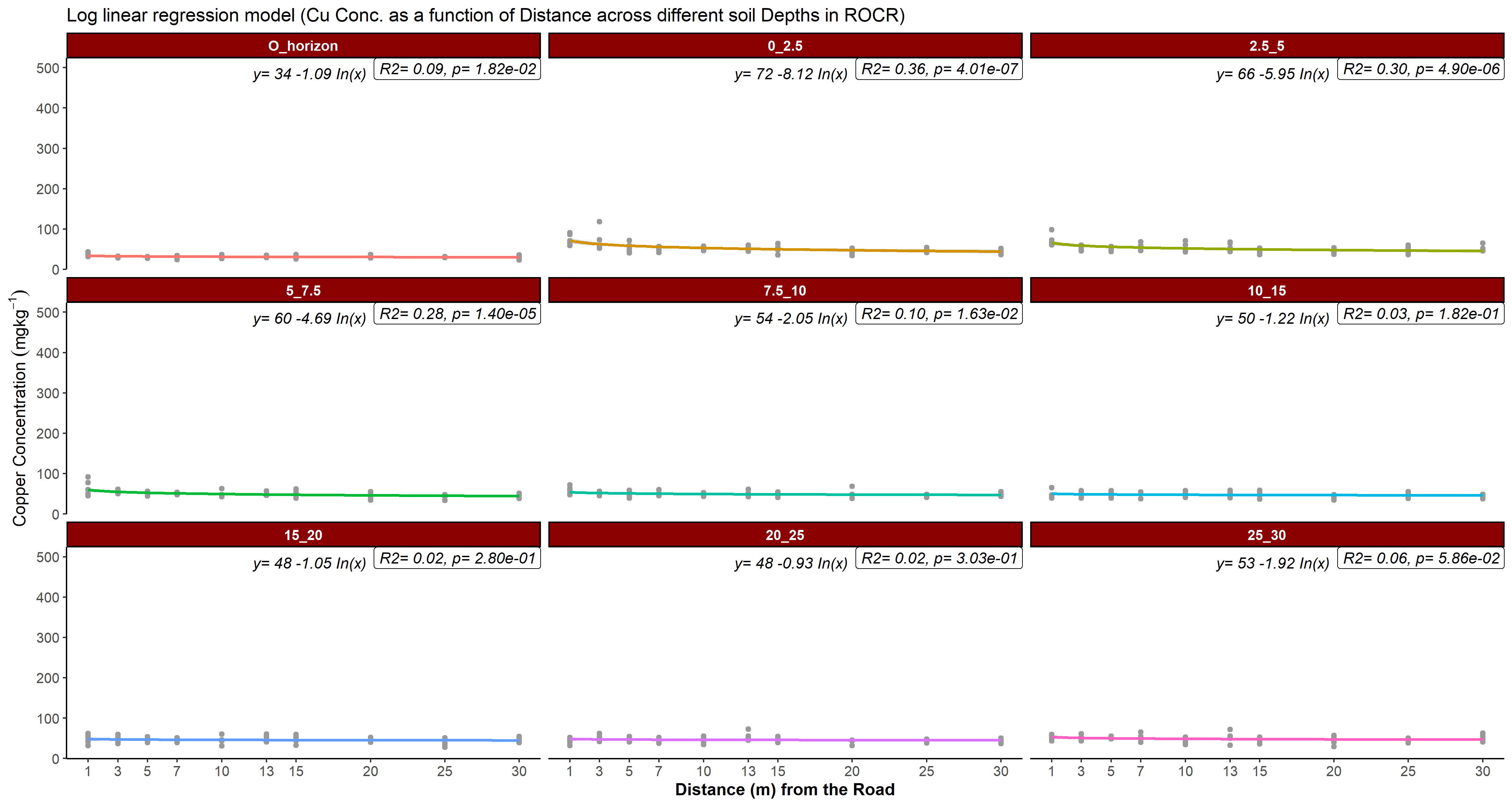

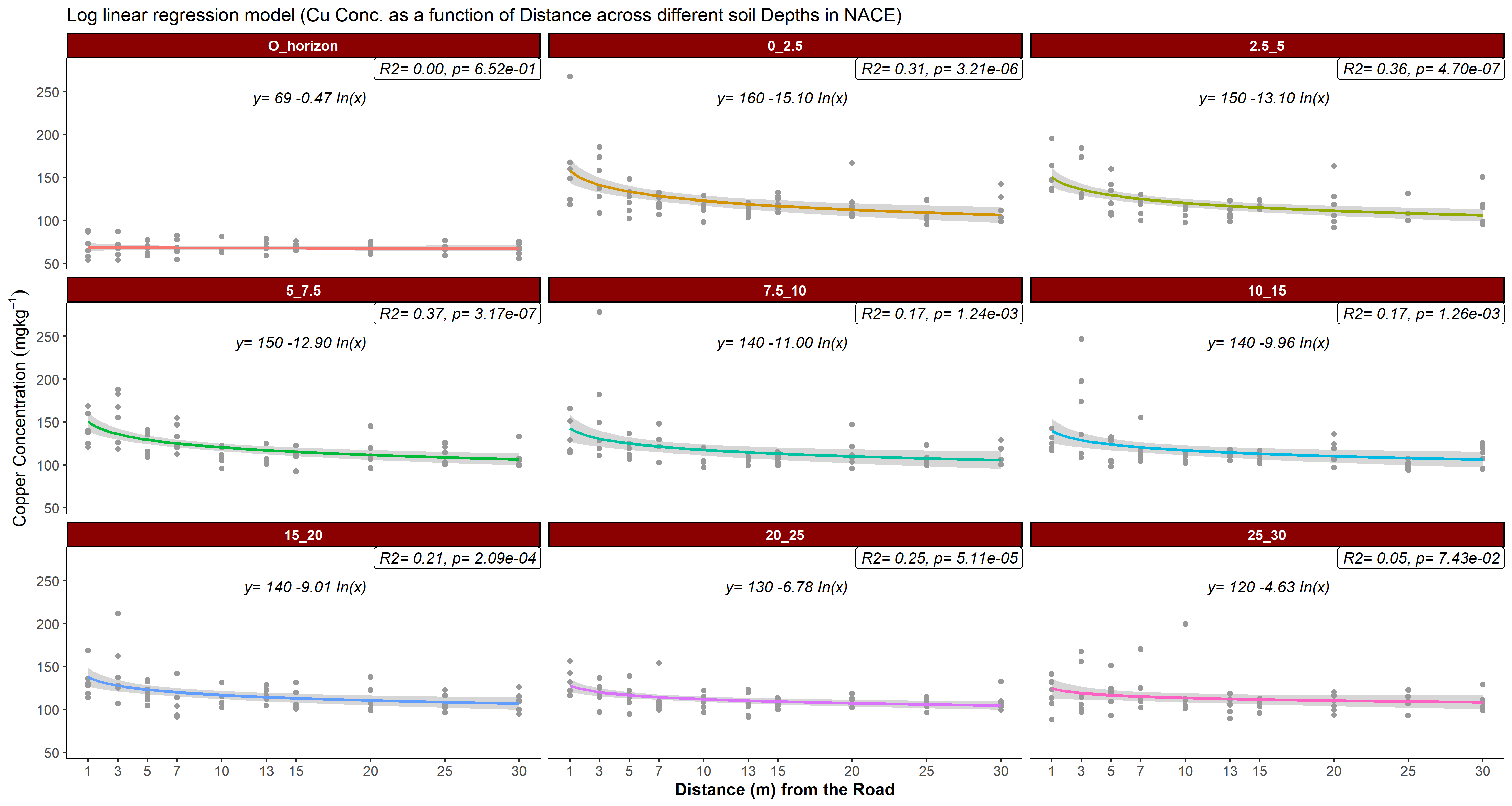


**Supplementary Figure 3.** Log linear regression model of between Cu concentration and road distance for each soil depth in Rock Creek National Park (ROCR) NACE (National Capital Parks-East).


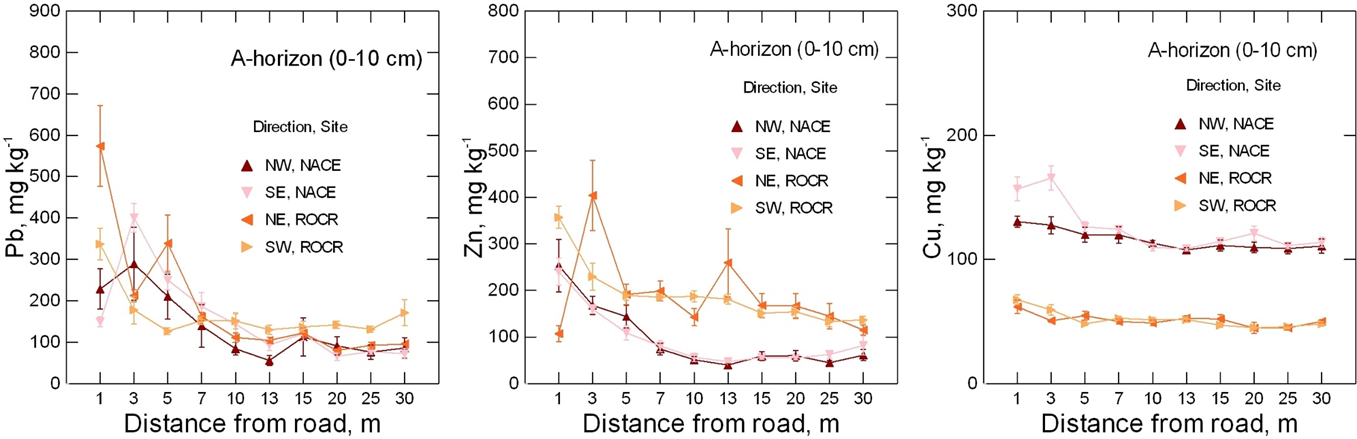


**Supplementary Figure 4.** Heavy metal concentrations in A-horizon (0-10 cm depth) at two sites as affected by distance from the road and the transect direction. The main prevailing winds are from the NW (see Fig. 1). Data points are means across four depth increments (0-2.5, 2.5-5.0, 5.0-7.5, and 7.5-10.0 cm) and four transects (for NW and SW directions) or two transects (for SE or NE directions). Bars are standard errors. The Distance x Direction x Site interaction effect was significant at P< 0.0001 for Pb and Zn and P<0.022 for Cu.
