# Supplementary material for: Soil lead, zinc, and copper in two urban forests as influenced by highway proximity
Source: J Environ Qual. 2024 Oct 21;54(1):275–88. doi: 10.1002/jeq2.20642 (PMC11718127; doi:10.1002/jeq2.20642)
Supplement: Supplementary file 2 — Supplemental Material [file JEQ2-54-275-s002.docx]

**Supplementary Table 1.** Selected chemical and physical properties of the soils in two National Park forests at eight depth increments: Average (SE) values for Bulk density, pH, Cation exchange capacity (CEC), Organic matter (OM), and Mehlich-3 extraction (M3).

| Soil Depth (cm) | BD  (g cm^-3^) | pH_water_ | OM  (g kg^-1^) | CEC  (cmol kg^-1^) | M3-P  (mg kg^-1^) | M3-Ca  (mg kg^-1^) | M3-Mg  (mg kg^-1^) | M3-K  (mg kg^-1^) |
| --- | --- | --- | --- | --- | --- | --- | --- | --- |
| National Capital Parks-East (NACE) | | | | | | | | |
| 0-2.5 | 1.40(0.02) | 3.85 (0.04) | 6.35 (0.46) | 6.40 (0.85) | 15.5 (1.06) | 141 (12.0) | 52.0 (5.66) | 32.5 (5.30) |
| 2.5-5 | 1.41(0.02) | 3.80 (0.00) | 4.6 (0.14) | 5.00 (0.07) | 13.5 (0.35) | 112 (1.06) | 37.5 (1.06) | 20.5 (1.06) |
| 5-7.5 | 1.46(0.02) | 3.85 (0.04) | 2.75 (0.25) | 3.80 (0.14) | 11.0 (0.71) | 89.5 (0.35) | 29.5 (0.35) | 16.5 (1.77) |
| 7.5-10 | 1.51(0.02) | 4.10 (0.14) | 1.95 (0.04) | 2.70 (0.49) | 7.50 (1.06) | 86.5 (2.47) | 25.0 (0.00) | 19.0 (4.24) |
| 10-15 | 1.51(0.02) | 4.25 (0.04) | 1.65 (0.18) | 2.10 (0.21) | 7.00 (0.71) | 83.5 (3.18) | 26.5 (1.77) | 16.5 (0.35) |
| 15-20 | 1.59(0.02) | 4.35 (0.04) | 1.15 (0.11) | 1.80 (0.00) | 5.50 (1.06) | 80.0 (1.41) | 22.0 (0.71) | 15.5 (3.18) |
| 20-25 | 1.63(0.01) | 4.45 (0.05) | 0.75 (0.18) | 1.45 (0.11) | 4.50 (0.35) | 75.5 (3.18) | 20.5 (1.06) | 15.0 (2.83) |
| 25-30 | 1.66(0.02) | 4.45 (0.11) | 0.85 (0.11) | 1.55 (0.18) | 18.5 (10.96) | 78.0 (1.41) | 21.0 (0.00) | 16.5 (2.47) |
| Rock Creek Park (ROCR) | | | | | | | | |
| 0-2.5 | 1.34(0.01) | 5.25 (0.25) | 7.15 (0.53) | 11.4 (1.77) | 22.0 (0.71) | 994 (303) | 291 (55.5) | 163 (14.9) |
| 2.5-5 | 1.37(0.02) | 4.8 (0.28) | 5.05 (0.25) | 10.2 (1.66) | 15.5 (3.18) | 659 (256) | 233 (65.8) | 140 (18.7) |
| 5-7.5 | 1.43(0.02) | 4.65 (0.18) | 4.10 (0.00) | 9.65 (1.87) | 16.0 (3.54) | 557 (214) | 202 (50.6) | 112 (3.89) |
| 7.5-10 | 1.45(0.02) | 4.70 (0.07) | 3.60 (0.21) | 7.20 (1.91) | 12.5 (1.06) | 424 (157) | 152 (41.0) | 104 (1.77) |
| 10-15 | 1.48(0.02) | 4.70 (0.14) | 2.85 (0.25) | 8.90 (3.25) | 12.5 (2.47) | 525 (249) | 215 (90.9) | 108 (10.3) |
| 15-20 | 1.54(0.02) | 4.65 (0.11) | 2.45 (0.18) | 7.20 (2.40) | 10.0 (1.41) | 391 (172) | 171 (68.9) | 93.0 (3.54) |
| 20-25 | 1.57(0.02) | 4.75 (0.05) | 2.25 (0.32) | 6.55 (1.66) | 7.50 (2.47) | 362 (120) | 162 (43.5) | 101 (6.01) |
| 25-30 | 1.61(0.02) | 4.80 (0.10) | 2.20 (0.42) | 5.95 (0.81) | 8.00 (2.83) | 324 (98.5) | 159 (33.2) | 94.0 (15.6) |

**Supplementary Table 2.** The total stock (kg ha^-1^) in two National Park forests of lead (Pb), zinc (Zn), and copper (Cu) in the O-horizon and mineral soil samples to 30 cm depth.

|  | Pb (kg ha^-1^) | | Zn (kg ha^-1^) | | Cu (kg ha^-1^) | |
| --- | --- | --- | --- | --- | --- | --- |
| Distance From Road (meter) | National Capital Parks-East **(NACE)** | | | | | |
|  | O-horizon | 0-30 cm | O-horizon | 0-30 cm | O-horizon | 0-30 cm |
| 1 | 0.11 | 991 | 0.36 | 763 | 0.17 | 619 |
| 3 | 0.64 | 1034 | 0.56 | 493 | 0.36 | 660 |
| 5 | 0.22 | 524 | 0.59 | 349 | 0.38 | 557 |
| 7 | 0.27 | 361 | 0.44 | 246 | 0.39 | 547 |
| 10 | 0.19 | 334 | 0.53 | 215 | 0.38 | 523 |
| 13 | 0.19 | 228 | 0.45 | 201 | 0.38 | 509 |
| 15 | 0.15 | 257 | 0.51 | 220 | 0.39 | 509 |
| 20 | 0.14 | 152 | 0.45 | 229 | 0.38 | 524 |
| 25 | 0.09 | 181 | 0.37 | 246 | 0.35 | 500 |
| 30 | 0.15 | 221 | 0.39 | 314 | 0.37 | 516 |
|  | Rock Creek Park **(ROCR)** | | | | | |
| 1 | 0.13 | 1077 | 0.27 | 828 | 0.08 | 241 |
| 3 | 0.05 | 482 | 0.37 | 839 | 0.13 | 230 |
| 5 | 0.04 | 576 | 0.52 | 731 | 0.11 | 221 |
| 7 | 0.05 | 432 | 0.56 | 725 | 0.13 | 221 |
| 10 | 0.03 | 371 | 0.47 | 669 | 0.12 | 212 |
| 13 | 0.06 | 348 | 0.46 | 755 | 0.14 | 231 |
| 15 | 0.05 | 389 | 0.43 | 633 | 0.13 | 214 |
| 20 | 0.06 | 348 | 0.54 | 610 | 0.14 | 196 |
| 25 | 0.04 | 314 | 0.33 | 561 | 0.12 | 199 |
| 30 | 0.06 | 468 | 0.36 | 536 | 0.14 | 210 |

**Supplementary Table 3.** Correlation between Earthworms and O- and A-horizon (0-10 cm soil) and average Biota-to-soil accumulation factor (BASF) at 3, 5, 10, and 30 m from the road in two National Park forests for lead (Pb), zinc (Zn), and copper (Cu). BASF means in a column followed by the same upper-case letter are not significantly different at p <0.05.

|  | National Capital Parks-East (NACE) | | | | Rock Creek Park (ROCR) | | | |
| --- | --- | --- | --- | --- | --- | --- | --- | --- |
|  | Correlation Earthworm with | | BSAF | | Correlation Earthworm with | | BSAF | |
|  | O-horizon | A-horizon | O-horizon | A-horizon | O-horizon | A-horizon | O-horizon | A-horizon |
| Pb | 0.36^*^ | 0.32^*^ | 3.86^A^ | 0.72^A^ | 0.36 | 0.32^*^ | 6.87^A^ | 0.37^A^ |
| Zn | 0.56^*^ | 0.33 | 4.2^A^ | 3.9^A^ | -0.21 | 0.43^*^ | 4.7^A^ | 1.7^B^ |
| Cu | 0.08 | -0.11 | 1.01^B^ | 0.51^B^ | 0.08 | 0.18 | 1.96^A^ | 1.1^A^ |

^*^Significant (p-value <0.05)
